# Deciphering the Structure and Formation of Amyloids in Neurodegenerative Diseases With Chemical Biology Tools

**DOI:** 10.3389/fchem.2022.886382

**Published:** 2022-05-12

**Authors:** Isabelle Landrieu, Elian Dupré, Davy Sinnaeve, Léa El Hajjar, Caroline Smet-Nocca

**Affiliations:** ^1^ University Lille, Inserm, CHU Lille, Institut Pasteur de Lille, U1167 - RID-AGE - Risk Factors and Molecular Determinants of Aging-Related Diseases, Lille, France; ^2^ CNRS EMR9002 Integrative Structural Biology, Lille, France

**Keywords:** amyloid fibril, aggregation, neurodegenerative diseases, protein semisynthesis, posttranslational modifications, native chemical ligation, fluorescent probes, nanobody

## Abstract

Protein aggregation into highly ordered, regularly repeated cross-β sheet structures called amyloid fibrils is closely associated to human disorders such as neurodegenerative diseases including Alzheimer’s and Parkinson’s diseases, or systemic diseases like type II diabetes. Yet, in some cases, such as the HET-s prion, amyloids have biological functions. High-resolution structures of amyloids fibrils from cryo-electron microscopy have very recently highlighted their ultrastructural organization and polymorphisms. However, the molecular mechanisms and the role of co-factors (posttranslational modifications, non-proteinaceous components and other proteins) acting on the fibril formation are still poorly understood. Whether amyloid fibrils play a toxic or protective role in the pathogenesis of neurodegenerative diseases remains to be elucidated. Furthermore, such aberrant protein-protein interactions challenge the search of small-molecule drugs or immunotherapy approaches targeting amyloid formation. In this review, we describe how chemical biology tools contribute to new insights on the mode of action of amyloidogenic proteins and peptides, defining their structural signature and aggregation pathways by capturing their molecular details and conformational heterogeneity. Challenging the imagination of scientists, this constantly expanding field provides crucial tools to unravel mechanistic detail of amyloid formation such as semisynthetic proteins and small-molecule sensors of conformational changes and/or aggregation. Protein engineering methods and bioorthogonal chemistry for the introduction of protein chemical modifications are additional fruitful strategies to tackle the challenge of understanding amyloid formation.

## 1 Introduction

Amyloids correspond to amorphous deposits of insoluble proteinaceous materials that are found in a variety of body tissues and organs. Amyloidoses is a group of diseases associated with amyloid deposits, including localized amyloidoses such as many neurodegenerative disorders (NDs) or type-II diabetes mellitus, and systemic amyloidoses. Amyloidoses can be also defined as “protein misfolding diseases” since their molecular basis relies on misfolding as an early event in the amyloid transformation (Benson et al., 2018; Benson et al., 2020). Indeed, the amyloid-forming protein converts into an abnormal, misfolded conformation from the same primary sequence that otherwise encodes either its native functional structure for globular proteins or its unfolded, dynamic conformational ensemble for intrinsically disordered proteins (IDPs) (Dobson, 2003; Knowles et al., 2014). Genetic alterations leading to protein misfolding may increase protein aggregation rate, modify mRNA splicing or impact the protein lifecycle. Moreover, changes in the oxidation state, posttranslational modification (PTM) patterns, protein interaction networks or environmental factors can trigger amyloid transformation without involvement of any mutation. Ultimately, protein misfolding results in a “loss-of-function” and/or a “gain-of-toxic function” (Winklhofer et al., 2008). However, amyloid formation cannot be strictly reduced to a defect of protein folding: defects in the cellular machinery of protein folding and quality control (mediated by molecular chaperones) or protein homeostasis (mediated by the proteasome and the lyzosomes) often associated to ageing are major players in the process. Cellular responses to external stimuli might in addition participate in the process by inducing incorrect protein trafficking, mislocalization, and aberrant interactions with aggregates or “seeds”. Mislocalization can give rise to changes in the oxidation state, in the PTM patterns or levels, and in the protein interaction networks (Yan et al., 2013; Ke et al., 2020).

**FIGURE 1:**
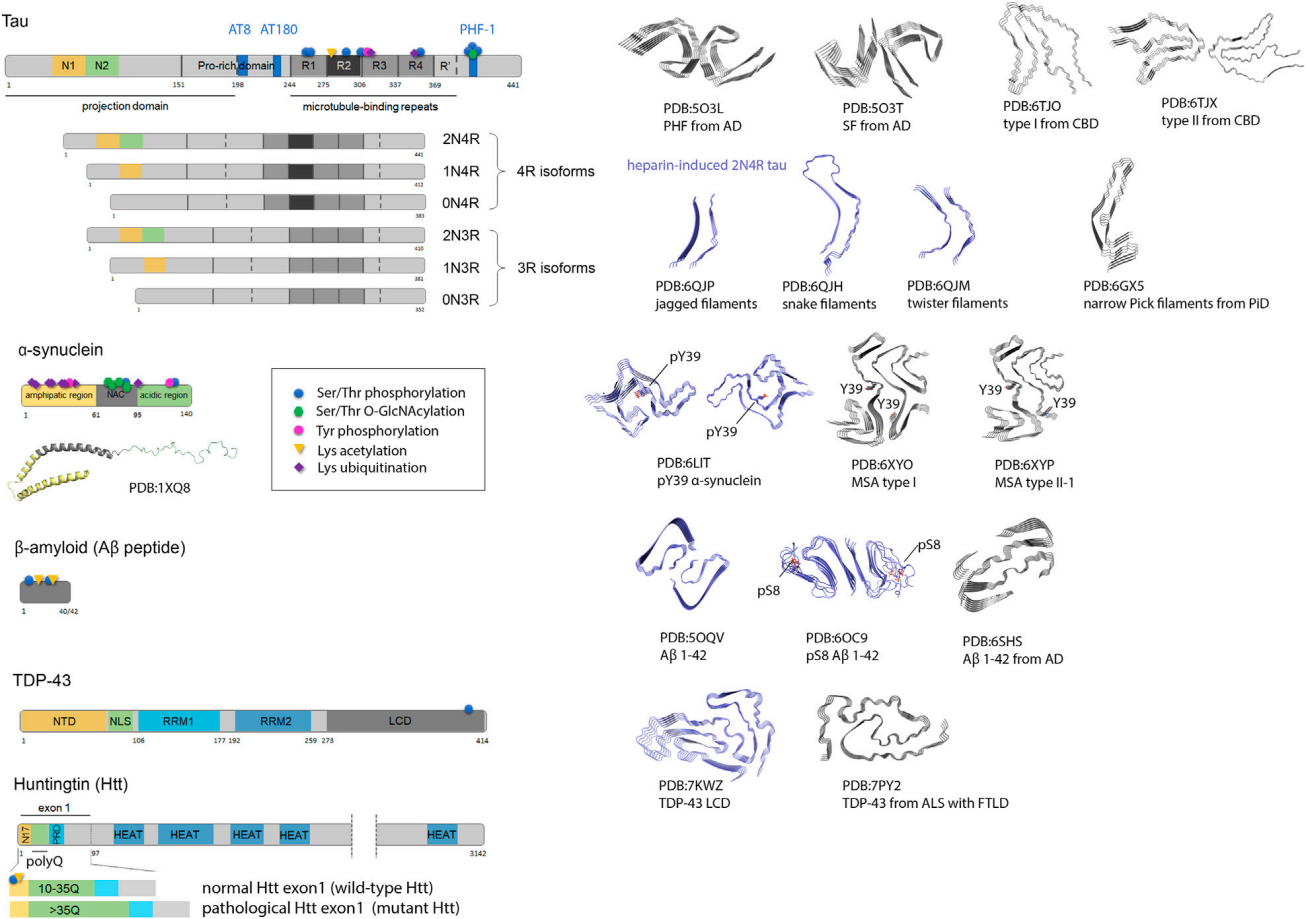
Amyloid proteins involved in neurodegenerative diseases (tau, Aβ, α-synuclein, TDP-43 and Huntingtin), and their amyloid folds and polymorphs. Scheme of protein sequences highlighting domains, and eventually isoforms are presented (left). For tau protein, some prominent pathological phospho-epitopes (AT8, AT180, PHF-1) associated to tauopathies are indicated. N1, N2, N-terminal inserts; R1-R4, microtubule-binding repeats 1 to 4; NAC, non-amyloid-β component; NTD, N-terminal domain; NLS, nuclear localization signal; RRM, RNA Recognition Motifs; LCD, low complexity domain; N17, 17-residue N-terminal region; PRD, proline-rich domain; polyQ, polyglutamine; HEAT, HEAT repeats. The solution NMR structure of micelle-bound α-synuclein is depicted. Some PTM sites studied by chemical ligation and/or chemical mutagenesis are indicated. The cryo-EM structures of representative fibrils from individual brains (grey structures) and synthetic fibrils (blue structures) highlight structural discrepancies pointing to the role of trans-acting cofactors and PTMs of amyloid proteins in the amyloidogenic process. For a more comprehensive description of the multiple tau folds associated to diverse tauopathies and their classification, refer to (Shi et al., 2021). AD, Alzheimer’s disease; CBD, corticobasal degeneration; PiD, Pick’s disease; MSA, multiple system atrophy; ALS with FTLD, amyotrophic lateral sclerosis with frontotemporal lobar degeneration.

Natively folded proteins involved in amyloidosis are for instance transthyretin (TTR) associated to familial amyloidotic cardiomyopathy, superoxide dismutase-1 (SOD-1) and transactive response DNA binding protein 43 (TDP-43) associated to familial amyotrophic lateral sclerosis (fALS), Huntingtin (Htt) associated to Huntington’s disease (HD), or the cellular form of prion protein PrP^C^ associated to prion diseases or transmissible spongiform encephalopathies (TSEs). The latter are the only transmissible neurodegenerative diseases identified to date arising from proteinaceous infectious particles (i.e., without involvement of any nucleic acid) called prions (PrP^Sc^) (Prusiner, 1982; Aguzzi and Calella, 2009). The IDP class includes the β-amyloid peptide (Aβ) as one of the sequential proteolysis products of amyloid precursor protein (APP) in Alzheimer’s disease (AD), tau protein in AD and other tauopathies, α-synuclein in Parkinson’s disease (PD), and islet amyloid polypeptide (IAPP) in type II diabetes (Figure 1). It should be noted that IDPs or alternatively long intrinsically disordered regions (IDRs), have generally important signaling and regulatory functions despite their disordered nature, acting as scaffolds for versatile interactions with multiple binding partners in cell signaling or to stabilize large structural components of the cells (Morris et al., 2011).

First histologically stained with Congo red, amyloids are widely detected with Thioflavin S (ThS) or Thioflavin T (ThT) fluorescent dyes that are used for both histological staining and the *in vitro* kinetics follow-up of amyloid assembly. In tissues, amyloids can be visualized under polarized light using the apple-green birefringent properties of the bound Congo red dye owing to its specific orientation in the regular arrangement of amyloid’s proteinaceous component (Howie and Brewer, 2009). For clinical use, aggregated amyloid imaging involves the search of radioligands for positron emission tomography (PET) that enable the noninvasive detection of amyloids in the brain with either pan-amyloids or, recently developed, selective imaging agents for diagnosis purpose like Pittsburgh compound B (PiB) targeting Aβ (Shin et al., 2011; Mathis et al., 2017).

In 1950s, electron microscopy shed light into their fibrillary structure while X-ray diffraction showed a typical pattern of structures dominating by β-sheet conformations. The fibrillar structure of amyloids is constituted by the assembly of a given protein -or part of it-as a repetition unit in a cross β-sheet conformation running perpendicular to the fibril axis (Figure 1). At low resolution as investigated by electron or atomic force microscopy, or by spectroscopic methods such as Fourier transform infrared spectroscopy or circular dichroism, amyloids from multiple origins share structural and spectroscopic similarities.

The investigations of amyloid structures at high resolution has benefited from the development of biophysical approaches such as X-ray crystallography, solid-state nuclear magnetic resonance (ssNMR) or more recently, cryo-electron microscopy (cryo-EM). The latter enables now to distinguish between diseases with close clinical features but associated to distinct strains (Shi et al., 2021). However, despite the abundance of structural and kinetics studies of amyloid proteins, external stimuli or stress signals as well as the molecular mechanisms that drive a native functional conformation to protein misfolding from the same primary sequence are not yet fully understood. Moreover, prediction of amyloid folds is still an issue since amyloid assembly is not limited to intramolecular contacts (Anfinsen et al., 1961; Anfinsen, 1973; Jumper et al., 2021; Tunyasuvunakool et al., 2021). Therefore, structure predictions based on protein sequence or evolution-based approaches could not be properly applied to protein aggregation and pathological amyloid fibrils that also make use of intermolecular interactions and are not evolutionarily selected (Pinheiro et al., 2021).

In view of the causative roles that the amyloids have in a large number of diseases with yet unmet medical need, understanding their formation and structure is a priority. This is also a challenge due their solid-like state, polymorphic nature, the multi-causative aspect of amyloidosis, the various organs and tissues affected by the process and the complex mechanistic steps of their formation. New tools issued from chemical biology are much needed to address these challenges in research, and open the way to innovative therapies.

## 2 Assembly, Properties and Propagation of Amyloid Fibrils in Neurodegenerative Diseases

### 2.1 Amyloid Structure and Formation

Amyloid fibrils are very stable protein assemblies at the thermodynamic (Buell et al., 2014) and mechanic (Knowles et al., 2007; Buehler and Cranford, 2010; Knowles and Buehler, 2011; Herling et al., 2015) levels due to the combination of both a tight packing of the polypeptide backbone into stacked β-sheets and intertwining of residue side chains. These cross-β structures result invariably in long, unbranched filaments. Hence, the amyloid core is stabilized by a wide array of non-covalent interactions and this packing by far exceeds the stability of the native 3D fold. However, hydrogen bonding interactions engaging the polypeptide main chain are prevalent explaining the structural similarity between amyloids despite the variety of protein sequences in sharp contrast with protein native state involving a great number of native contacts between side chains of key residues (Fandrich, 2002; Dobson, 2003). Despite this apparent simplicity, amyloid can take a large number of distinct folds illustrating their intricate structures and remarkable heterogeneity. Unexpectedly, an identical sequence can adopt several folds called polymorphs or strains, associated with different diseases, and even coexist in a single disease such as tau protein or Aβ42 in AD (Fitzpatrick et al., 2017; Yang et al., 2022).

Amyloid structures are only a single, yet peculiar, class of aggregates that can build up from a misfolded or denatured protein that stems either from an IDP or an initially folded protein for which secondary structures were reassigned. As every conformer, their formation depends on both thermodynamics (relative free energy) and kinetics parameters (interconversion rates) that confer stability (Baldwin et al., 2011; Buell et al., 2014). In addition, tight packing of the amyloid core is responsible for a lack of polypeptide chain accessibility to degradation (Novak et al., 1993) that confer to amyloids an increased lifetime (Makin et al., 2005). Amyloid formation mainly consists of three main stages defined as nucleation, growth and maturation. During the nucleation step, metastable species expose aggregation-prone sequences that self-associate into soluble oligomers or amorphous aggregates that serves as nuclei of the fibrillization process. Then, the fibrils rapidly grow during the elongation phase by an autocatalytic mechanism involving the addition of new monomers to the nuclei or seeds that convert their conformation into the templated amyloid fold resulting in insoluble, ordered structures with fibril-like morphologies. Prefibrillar aggregates or seeds evolved into fibrils or eventually, may be involved in amplifying aggregation through a secondary nucleation mechanism that catalyzes fibril assembly at the seed surface or upon fibril fragmentation (Cohen et al., 2013; Meisl et al., 2014; Gaspar et al., 2017; Rodriguez Camargo et al., 2021). This process was described as secondary nucleation since it requires the formation of protofibrils or pre-fibrillar species to be effective. Finally, during the fibril maturation, the protofilaments formed during the growth phase associate through protein-protein interactions at the protofilament interfaces to form high-ordered fibrillar structures as seen by electron or atomic force microscopy. In cells, the maturation step may involve other proteinaceous components and PTMs including proteolysis leading ultimately to fibrillar deposits (Kimura et al., 1996; Braak et al., 2006; Aragão Gomes et al., 2021). The protofilaments can thus assemble into diverse interfaces leading to different morphologies or strains in the eye of the electron microscope. Some of them are periodic structures such as Paired Helical Filaments (PHFs) observed in AD by negative staining transmission electron microscopy (TEM) in which a pair of protofilaments assemble by twisting around each other with a helical turn period of about 80 nm (Kidd, 1963).

Very recently, cryo-EM, remarkably exploited by Goedert, Scheres and collaborators, provides structural details at near-atomic resolution on amyloid folds of tau protein either from patients with various tauopathies or made *in vitro* with heparin as aggregation inducer (Fitzpatrick et al., 2017; Falcon et al., 2018a; Falcon et al., 2018b; Zhang et al., 2019a; Falcon et al., 2019; Zhang et al., 2020) highlighting profound structural discrepancies. The same has been noted for fibrils of α-synuclein, wild-type or mutants, in synucleinopathies (Li et al., 2018a; Li et al., 2018b; Guerrero-Ferreira et al., 2018; Guerrero-Ferreira et al., 2020; Schweighauser et al., 2020) or TDP-43 in ALS/FTD (amyotrophic lateral sclerosis/frontotemporal dementia) (Arseni et al., 2021) and synthetic filaments. Each new fibril structure solved by cryo-EM from different diseases highlights the incredible plasticity of protein sequences of various origins to adopt multiple amyloid folds as well as the complexity of cofactors that shape the final amyloid structure and the misfolding pathway (Diaz-Espinoza, 2021). In the case of tau protein, the involvement of several protein isoforms in patients’ fibrils (either by a combination of the 3R/4R isoforms, as in AD, or exclusively 3R, as in Pick’s disease, or 4R isoforms, as in corticobasal degeneration) is in part responsible of distinct folds of tau in tauopathies. Interestingly, the amyloid folds of tau between distinct tauopathies are different, but individuals with the same disease share an identical fold. Actually, tau folds into either three or four layers when embedded in filaments, each categories being divided into distinct folds thereby suggesting a possible hierarchical classification of diseases based on tau amyloid folds (Shi et al., 2021).

Remarkably, in most instances, the region encompassed within the fibril core only represents part of the protein sequence while a large, if not the most part of the protein, retains a high degree of flexibility and accessibility, and projects from the fibril core. For example, in the case of tau protein, the ratio of residues involved in the fibril core can be as low as roughly 20% of the largest isoform (Fitzpatrick et al., 2017; Falcon et al., 2019; Zhang et al., 2020; Shi et al., 2021). The remaining of the sequence, i.e. the N- and C-terminal segments, forms a “fuzzy coat” around the amyloid core (Wegmann et al., 2013). First suggested by negative-staining and scanning TEM (Wischik et al., 1988), the structurally variable regions of tau fibrils largely escape further structural characterization by cryo-EM or ssNMR (Andronesi et al., 2008). They can still be addressed by ensemble-based methods such as solution-state NMR (Sillen et al., 2005; Bibow et al., 2011; Lippens et al., 2016) as well as fluorescence-based single-molecule approaches that enables dissecting low-populated, transient states that form during the amyloid assembly including oligomers or secondary nucleation processes (Kundel et al., 2018a; Kundel et al., 2018b; Kjaergaard et al., 2018; Rice et al., 2021; Yang et al., 2021). These structurally dynamic, disordered regions yet deserve particular attention as they can play a regulatory role in amyloid fibril formation especially through their abundant and diverse PTMs including truncations (Morris et al., 2015; Despres et al., 2017).

Cryo-EM has become prominent for the structural analysis of macromolecules from *postmortem* tissues at a near-atomic resolution (Figure 1). Cryo-EM structures point toward a role of cofactors and PTMs in fibril assembly and polymorphism (Li and Liu, 2021). Indeed, unresolved, non-proteinaceous densities (Falcon et al., 2019; Schweighauser et al., 2020; Zhang et al., 2020; Arseni et al., 2021; Shi et al., 2021), or (poly)ubiquitin chains (Arakhamia et al., 2020) were found in the vicinity of fibril surfaces made of tau, α-synuclein or TDP-43 proteins. Beyond their potential participation to the amyloidogenic transformation, these protein and non-protein entities highlight interfaces that could be targeted to prevent fibril assembly or disrupt existing fibrils. Even though atomic details were not observed for PTMs corresponding to the addition of small chemical groups such as Ser/Thr/Tyr phosphorylation, or Lys acetylation and methylation, these modifications are definitely present in fibrillar structures extracted from patient brains as detected by MS methods although heterogeneity or location outside the fibril core in adjacent flexible regions correspond to weaker densities or no density at all in cryo-EM high-resolution maps leaving open the role of these PTMs in fibrillogenesis, fibril packing and stabilization, and polymorph selection. Finally, the structure of the amyloid fibril as a final product provide no clue about the mechanism and kinetics as well as the intermediate structures that appears along the fibrillization process.

### 2.2 Features and Properties Related to Amyloids

Mature fibrillar assemblies, as histopathological hallmarks of amyloidoses, have long been considered as the causal agent of disease pathogenesis. In AD, a toxic “gain-of-function” is tightly related to the formation of amyloid structures that at least partly correlates with clinical manifestations when a certain amount of aggregates and/or certain brain areas were affected (Chung et al., 2018). It has also become clear that small aggregates formed early during the fibrillization process, referred to as oligomers or prefibrillar species, would be the most toxic species and more damaging to neurons than fibrils (Caughey and Lansbury, 2003). Hence, it has been argued they are most probably best targets than fibrillar species from a therapeutic perspective. However, as metastable assemblies of heterogenous composition and structure, their molecular description with experimental methods are scarce. Atomic details of amyloid structures from combination of experimental models (ssNMR, X-ray, AFM, TEM) were complemented by molecular dynamics simulations. Simulations provide details at different aggregation stages of amyloid peptides such as Aβ40/42, tau, α-synuclein ranging from the monomeric to the oligomeric states and protofibrils up to amyloid fibrils (Nasica-Labouze et al., 2015; Ilie and Caflisch, 2019; Nguyen et al., 2021). For example, structures of low-populated intermediates of Aβ or IAPP were trapped in NMR or X-ray studies and observed in computational studies. Such models provide a molecular basis for pharmacological targeting of early, on-pathway aggregation species. While experimental models give time- and space-averaged properties, computational models offer a view of dominant states in the aggregation pathway by sampling various time and length scales and using different representations such as the all-atom, the coarse-grained and mesoscopic models. The major issues relate on the accuracy of the force field, the concentration of monomers, and the limited size of the simulated system. Moreover, the simulation time is several orders of magnitude less than the time of *in vitro* or *in vivo* fibrillar assembly that takes typically several hours up to several days. Besides the investigations of the early steps of aggregation and the mechanism of fibril elongation, interactions with cell membrane or metal ions, the role of PTM and complex coacervation (e.g., for tau protein) can also be explored with computational methods as discussed in comprehensive reviews (Nasica-Labouze et al., 2015; Ilie and Caflisch, 2019; Nguyen et al., 2021). Furthermore, a crosstalk between an amyloid conformation, or strain, and a naive amyloid-prone protein is a critical event in neurodegenerative diseases (Soto and Pritzkow, 2018). The ability of amyloid to convert normal protein conformers into new amyloid conformations is a process coined as seeding in which “seeds”, an elusive term with respect to their composition and structure, act as templates of their self-copy. Hence, the main property of seeds is the imprinting of the misfolded conformation as a “conformational memory” that may be structurally propagated over several seeding generations (Frost et al., 2009; Nizynski et al., 2018). This is another aspect in favor of the irreversible cellular accumulation of amyloids once they have started forming. Moreover, the amyloid transformation of a given protein could also be triggered in a process named “cross-seeding” by heterologous seeds, i.e., seeds formed by a heterologous protein, be it from an unrelated protein, another isoform or a mutant form of the same protein. To implement innovative therapeutic routes in amyloid diseases, understanding the mechanisms of amyloid formation and emphasizing critical molecular species along the pathway are of the highest importance.

According to Braak staging, proteinaceous lesions of PD and AD progress through the brain in a spatiotemporal manner (Braak and Braak, 1991; Braak and Braak, 1995; Braak et al., 2004). They first start at defined, selectively vulnerable brain sites depending on the pathology, and gradually extend to neighboring neurons and distant brain structures through connected neurons. Overall, these pathologies progress silently over years before becoming symptomatic. Hence, it has been suggested that spreading of pathological amyloid species by cell-to-cell transmission is not confined to the sole prion proteins (Prusiner, 1982). By templating their own replication, many misfolded proteins including α-synuclein (Luk et al., 2012), β-amyloid (Ruiz-Riquelme et al., 2018), tau (Braak and Del Tredici, 2018) and huntingtin (Pearce and Kopito, 2018) behave like infectious prions by propagating seeds of various structures from a donor cell in a so-called “prion-like” spreading that results in the formation of amyloid aggregates in recipient cells (Mudher et al., 2017). However, the “prion-like” spreading hypothesis is still controversial and the spatiotemporal evolution of AD and PD that was elegantly demonstrated by Braak and colleagues is not a proof *per se* in favor of a prion-like spreading mechanism that physically involves seed release and capture from diseased to connected neurons. A model of selective neuron vulnerability has been proposed as an alternative to the “prion-like” spreading hypothesis arguing that neurons bearing aggregates or oligomers induce external stress on selected neuron populations that start producing aggregates in response to adverse stimuli (Walsh and Selkoe, 2016; Chung et al., 2018). The latter hypothesis does not preclude the “prion-like” spreading hypothesis, and both mechanisms may coexist.

Among the challenges attributed to amyloid diseases, the definition of the pathogenic species and how they can cross cellular membranes and spread from cell to cell is of crucial importance to be able to decipher disease progression and mechanisms, find specific and early diagnostic tools and devise efficient therapies (Colin et al., 2020). Linked to the prion-like propagation hypothesis, the concept of amyloid strains has evolved to explain distinct patterns of neuropathology and transmission through the central nervous system (CNS). The exact paths of cell-to-cell transmission of the pathogenic species seem not to be unique and might be dependent on the protein of interest. Nonetheless, this transmission is described as a non-cell-autonomous progressive spreading in many studied cases.

Finally, it was suggested that liquid-liquid phase separation (LLPS) that forms membraneless organelles by molecular reversible self-assembly, might be the missing link between protein misfolding, aggregation and pathogenesis associated to neurodegenerative disorders (Nedelsky and Taylor, 2019; Babinchak and Surewicz, 2020; Alberti and Hyman, 2021). Notably, many amyloidogenic proteins are prone to phase transition that can initiate protein misfolding and aggregation, and modulate their biological function as shown for tau (Ambadipudi et al., 2017; Zhang et al., 2017a; Hernández-Vega et al., 2017; Wegmann et al., 2018; Boyko et al., 2019; Majumdar et al., 2019; Kanaan et al., 2020; Singh et al., 2020; Rai et al., 2021), TDP-43 (Li et al., 2018c; Wang et al., 2018; Babinchak et al., 2019; Conicella et al., 2020; Watanabe et al., 2020; Dang et al., 2021; Grese et al., 2021; Hallegger et al., 2021; Pakravan et al., 2021), α-synuclein (Sawner et al., 2021), the amyloidogenic type II diabetes-associated IAPP (Pytowski et al., 2020) and the fused in sarcoma (FUS) protein (Patel et al., 2015; Monahan et al., 2017; Murthy et al., 2019; Ishiguro et al., 2021; Levone et al., 2021; Reber et al., 2021). Understanding the molecular mechanisms of aberrant phase separation should provide new strategies to control protein aggregation in neurodegeneration.

### 2.3 Modulators of Amyloid Aggregation

#### 2.3.1 Trans Acting Factors of Protein Aggregation

The deposits and inclusions in neurodegenerative disorders such as NFTs, senile plaques, Lewy bodies… consist of several proteins and non-proteinaceous components (carbohydrates, nucleic acids, metals, lipids, lipid rafts and cholesterol) that could be linked to the amyloid polymorphisms observed in diseases (Kollmer et al., 2016; Stewart et al., 2016; Shahmoradian et al., 2019; Li and Liu, 2021). The proteome analysis of amyloid deposits has revealed hundreds of proteins (Drummond et al., 2017; Lutz and Peng, 2018). Some of them act as critical regulators in protein misfolding diseases exemplified by heat shock proteins (HSPs) and their co-chaperones, 14-3-3 proteins (Xu et al., 2013; Jia et al., 2014), S100B calcium-binding protein (Moreira et al., 2021), and the peptidyl-prolyl isomerases (PPIases) FKBPs and Pin1 (Hamdane et al., 2002; Landrieu et al., 2006a; Balastik et al., 2007; Lippens et al., 2007; Chambraud et al., 2010; Giustiniani et al., 2012; Giustiniani et al., 2014; Kamah et al., 2016; Chen et al., 2018; Wang et al., 2020a). Notably, Pin1 and 14-3-3 proteins interact with phosphorylated forms of tau making the link with amyloid PTMs (Lu et al., 1999; Zhou et al., 2000; Smet et al., 2004; Lim and Ping Lu, 2005; Smet et al., 2005; Pastorino et al., 2006; Landrieu et al., 2011; Kondo et al., 2017; Neves et al., 2021). A role of cofactors has been shed into light in the aggregation of tau protein to specifically address the challenges of forming amyloid fibrils from full-length tau *in vitro* (Fichou et al., 2018; Fichou et al., 2019) in contrast to Aβ or α-synuclein that readily form amyloid fibrils in a wider range of conditions. Glycosaminoglycans, lipid membranes and metal ions are key cofactors that were pointed out in the amyloidogenic process. They have been found to modulate aggregation rates and are associated with amyloid deposits within the brain. Interactions of amyloidogenic species with cofactors may represent an orthogonal strategy of intervention to aggregation inhibitors in neurodegenerative disorders.

#### 2.3.2 Posttranslational Modifications of Amyloid Proteins

**FIGURE 2:**
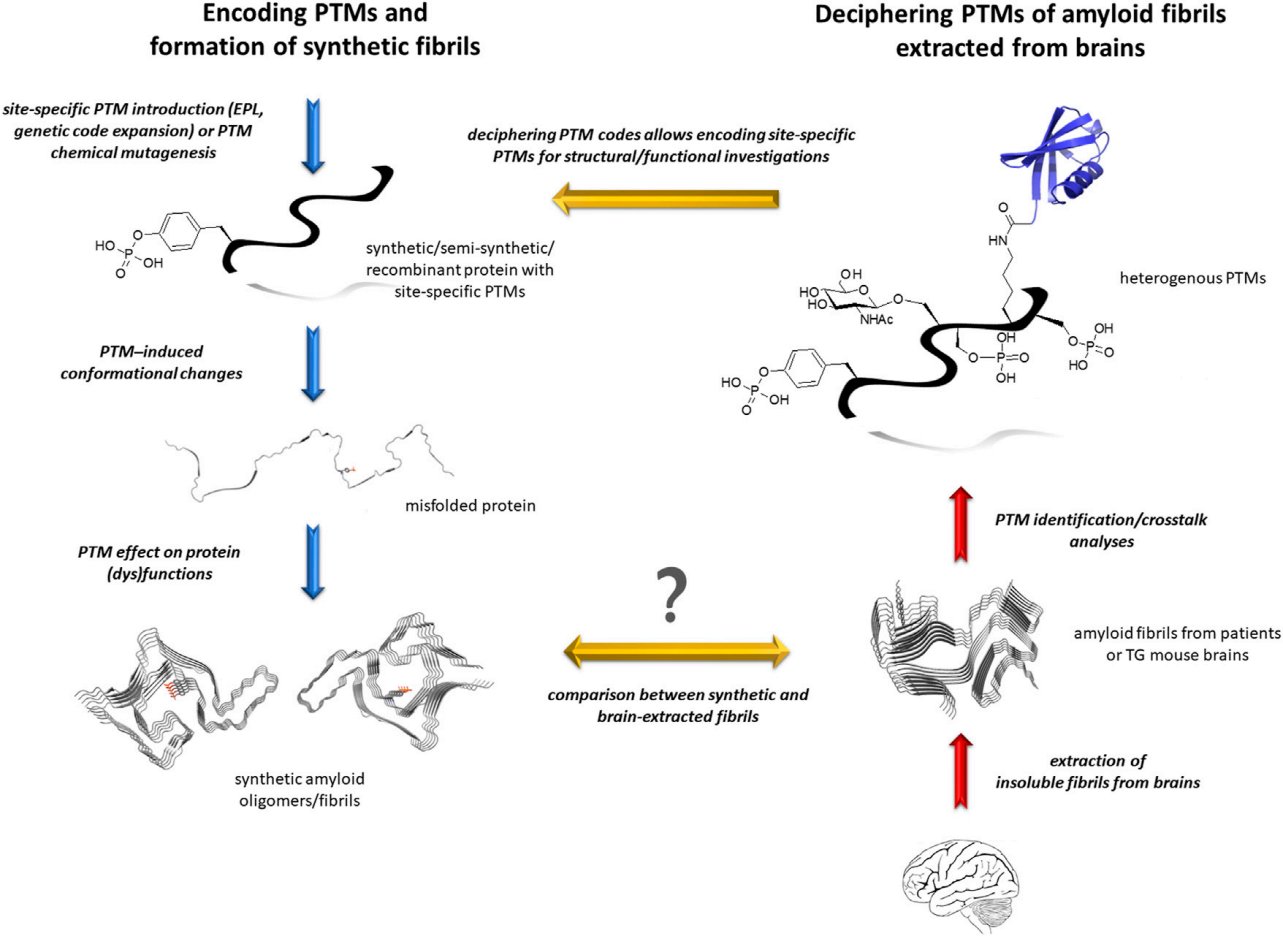
Investigating the role of PTMs in amyloidogenesis by protein semisynthesis and/or chemical mutagenesis. Amyloid proteins are extracted from patient brains and purified as insoluble material, then the PTM patterns of amyloid fibrils are deciphered by complementary biophysical and biochemical tools (right panel). The precise role of site-specific PTMs is unraveled by encoding individual PTM or pathological epitope using chemical biology tools. This allows investigating the effect of these specific chemical modifications on protein conformation and function, on oligomer and amyloid assembly, stability and properties (left panel). The synthetic fibril morphology and cryo-EM atomic structures could thus be compared to *bona fide* fibrils from patient brains, here illustrated with pY39 α-synuclein synthetic fibrils (PDB ID: 6LIT) and α-synuclein fibrils from MSA (PDB ID: 6XYO), providing important information about the role of cofactors in amyloid aggregation.

The polymorphism of amyloid structures from the same protein reflects distinct environments leading ultimately to different diseases. In this respect, PTMs and non-amino acid components associated with the fibrils have focused particular attention (Figure 2). Arising from the most recent cryo-EM structures of human prion PrP, wild-type α-synuclein from multiple system atrophy (MSA), tau from corticobasal degeneration (CBD) - all of which were from brains of patients- and phospho-Tyr39 (pY39) α-synuclein from semisynthesis (Arakhamia et al., 2020; Wang et al., 2020b; Schweighauser et al., 2020; Zhang et al., 2020; Zhao et al., 2020), it has been proposed to categorize PTMs based on their location with respect to the fibril core (Li and Liu, 2021). PTMs in the interior of the core are likely involved in the initial step of fibril assembly while PTMs on the exterior may act rather in the polymorph selection either by driving the folding of protofilaments or stabilizing the protofilament interface. The role in fibril assembly of PTMs outside the core, within the “fuzzy coat”, still remains poorly defined although they are known to regulate the protein functions, interactions and aggregation properties by modulating the rate of fibrillar assembly, toxicity and phase separation. Importantly, they remain accessible even within the amyloid fibril and may still be targeted by posttranslational modifying and proteolytic enzymes (Wegmann et al., 2013; Ulamec et al., 2020).

Hyperphosphorylation, a common feature of tau proteins in NFTs and inclusions from diverse tauopathies, is mainly found within the N- and C-terminal regions flanking the amyloid core (Morishima-Kawashima et al., 1995a; Hanger et al., 1998; Alonso et al., 2001; Hanger et al., 2007; Hanger et al., 2009; Šimić et al., 2016) that greatly inhibit the formation of filaments (Abraha et al., 2000; Lövestam et al., 2021). Tau also exhibits a large diversity of PTMs such as lysine ubiquitination, SUMOylation, acetylation and methylation (Morris et al., 2015; Wesseling et al., 2020). These modifications can be specifically linked to the disease stage and mediate the structural diversity of tau strains (Arakhamia et al., 2020). Interestingly, the seeding activity of the hyperphosphorylated, oligomeric tau species was found to be heterogeneous from one patient with pure, typical AD to another while enhanced seeding activity and worse clinical outcomes both correlate with specific PTM sites (Dujardin et al., 2020). Whereas phosphorylation of α-synuclein at serine 129 (pS129) is a dominant characteristic of PD inclusions such as Lewy bodies, its role in aggregation and toxicity of α-synuclein has not yet been clearly established (Anderson et al., 2006; Oueslati, 2016; Ghanem et al., 2022). TDP-43, the main component of intracellular ubiquitin inclusion bodies found as a hallmark of ALS-FTLD (Frontotemporal Lobar Degeneration), is hyperphosphorylated and polyubiquitinated whereas these PTMs were not detected in normal brain (Dong and Chen, 2018).

Commonly associated to phosphoproteins, the *O*-β-linked N-acetylglucosaminylation (*O*-GlcNAc) is another PTM of serine/threonine residues that corresponds to the addition of a single sugar moiety that is regulated in a dynamic fashion by the antagonist action of two enzymes, the *O*-GlcNAc transferase (OGT) and *O*-GlcNAc hydrolase (OGA) (Iyer and Hart, 2003). Protein *O*-GlcNAcylation is extremely sensitive to glucose uptake and metabolism that may be altered in aging brain. Additionally, the *O*-GlcNAc modification has been reported for neuronal proteins such as APP, tau and α-synuclein highlighting a potential role in neurodegenerative diseases (Lazarus et al., 2009; Ma et al., 2017). *O*-GlcNAcylation of amyloid-forming proteins has been shown to regulate aggregation (Yuzwa et al., 2014a) and to some extent, phosphorylation of tau (Liu et al., 2004; Gong et al., 2006; Liu et al., 2009; Smet-Nocca et al., 2011; Bourré et al., 2018; Cantrelle et al., 2021). The treatment of transgenic mice with Thiamet-G, an OGA inhibitor, results in increased brain *O*-GlcNAc levels, and alleviate tau pathology and associated neurodegeneration offering an alternative therapeutic strategy to kinase and aggregation inhibitors in tauopathies (Yuzwa et al., 2008; Yuzwa et al., 2012; Yuzwa et al., 2014b; Graham et al., 2014; Hastings et al., 2017; Lee et al., 2021).

#### 2.3.3 Proteolysis

In addition to PTMs involving the covalent linkage of proteinaceous or small chemical entities, truncated forms of amyloid proteins are also frequently found associated to pathological transformation. The N-terminal region of Htt is the site of HD-associated pathogenic changes through an elongation of the CAG repeat of *htt* gene encoding an expanded polyglutamine repeat. The truncated N-terminal proteoforms are more toxic than full-length Htt and form intranuclear inclusions that disrupt synaptic and axonal functions (Sun et al., 2002). At the basis of the amyloid cascade hypothesis in AD, the Aβ peptide is the product of the sequential cleavage of the transmembrane APP protein by secretases that generates peptides of different length, the most common forms being Aβ40 and Aβ42. With a proportion significantly increased in AD brain, this latter form is the most neurotoxic and readily form oligomers and fibrils in a wide range of conditions (Nirmalraj et al., 2020; Yang et al., 2022). A large panel of tau fragments resulting from cleavage at N- or C-terminal regions or both are found in fibrillar structures, cerebrospinal and interstitial fluids, and plasma of patients with different tauopathies (Quinn et al., 2018; Boyarko and Hook, 2021). The predominant role of proteolysis in various neurodegenerative disorders deserves particular attention for characterizing the fragments, their toxicity related to aggregation and transcellular spreading, and their role in the selection of strain polymorphs. This approach could afford new biomarkers and disease-modifying therapies by modulating the fragment generation and associated toxicity (Rodriguez Camargo et al., 2021).

The wide range of disease-associated modifications represents several challenges: 1) discriminating between physiological and disease-associated changes, 2) characterizing PTMs in terms of site-specific identification, quantification, and crosstalk between PTMs, and their relevance to disease, 3) characterizing PTM-induced conformational changes, 4) identifying enzymes responsible of the installation/removal of specific PTMs and defining the pathway of their (dys)regulation, 5) determining the functional role of specific PTMs in physiology and pathology, and 6) identifying and deciphering the role of amyloid interacting entities or cofactors (Kametani et al., 2020). The role of proteins, non-proteinaceous entities and PTMs in the aggregation process, toxicity and spreading of various species that form during the fibrillization course still deserves further investigations (Figure 2). They may have prominent implications in modulating nucleation, aggregation rate, selection of fibril polymorph, seeding capacity, and amyloid toxicity. Additionally, interactions of amyloid-prone proteins with cofactors and posttranslationally modifying enzymes could be valuable targets for therapeutic intervention (Dujardin et al., 2020).

## 3 Deciphering the Posttranslational Modification Codes of Amyloids: Combining Protein Engineering With the Chemical Biology’s Tool Kit

As small chemical groups or proteinaceous components, PTMs is a dynamic way to modulate physicochemical properties and hence, the biological and pathological functions of proteins by rapidly and reversibly enlarging the proteome complexity. In this regard, PTMs regulate the aggregation propensity of amyloid proteins, the stability of oligomers and seeds, the propagation of seeds and other toxic species, demixing into liquid droplets, … all as crucial steps in amyloidogenesis. Defining a PTM signature may be relevant to track disease-associated changes, connect changes in PTM patterns to a loss or gain of function, and find new biomarkers and therapeutic targets in disease-modifying strategies. Deciphering the role of site-specific PTMs is of highest importance in this area but this knowledge needs to overcome the issue of multiple, heterogenous modifications found in a cellular environment or provided *in vitro* by enzymatic activities. Mutation of site-specific positions, e.g. into alanine, is commonly employed to reduce the number of PTM sites (Despres et al., 2017). Introducing amino acids mimicking the physiochemical properties of posttranslationally-modified residues, such as aspartate or glutamate for phospho-serine/threonine, or glutamine for acetyl-lysine, is an easy way to achieve homogenous levels of modification but is poor proxy of the corresponding PTM (Paleologou et al., 2008). The alternative modification of proteins by the enzymatic route provides heterogenous patterns due to multiple sites, PTM crosstalk and different stoichiometry that are invariably associated to sample complexity for modified proteins (Theillet et al., 2012). This feature has been extensively described by our group illustrating the exceptional complexity of PTMs and PTM crosstalk within tau protein, as well as their impact on tau conformation and physiopathological functions (Landrieu et al., 2006b; Amniai et al., 2009; Landrieu et al., 2010; Leroy et al., 2010; Landrieu et al., 2011; Kamah et al., 2014; Lippens et al., 2016; Despres et al., 2017; Gandhi et al., 2017; Bourré et al., 2018; Despres et al., 2019; Cantrelle et al., 2021).

Chemical biology on the other hand provides a wide range of tools to unravel the role of PTMs in the mechanism of amyloid aggregation and tackle the process of fibril assembly. This goes hand-in-hand with progresses in protein engineering. Specifically, the development of efficient expression vectors combined to bacterial strains and other heterologous systems for recombinant protein expression, together with the use of multiple purification tags allowed the production of milligram amounts of proteins (depending on expression systems) with a high degree of purity. Although this procedure can be routinely implemented for the preparation of proteins, it is limited to the 20 genetically-encoded amino acids, excluding in most instances the possibility of chemical modifications of amino acids including insertion of PTMs, probes, or the incorporation of unnatural or d-amino acids. However, solving inherent limitations of site-specific modification of recombinant proteins has benefited from the development of both protein synthesis by chemical ligation strategies (Dawson et al., 1994; Moon et al., 2021) and genetic code expansion through reassignment of sense and nonsense codons combined to engineered aminoacyl-tRNA synthetase/tRNA pairs (Wang et al., 2001). In this area, cell-free expression systems are efficiently developed for the incorporation of unnatural amino acids (UAAs) that are not genetically encoded (Gao et al., 2019) or manipulating isotopic labeling schemes (uniform labeling, selective labeling and site-speciﬁc labeling) for NMR structural analyses. By controlling the isotopic scheme of amino acids used in cell-free reactions, these approaches allow reducing isotopic scrambling. This strategy has been successfully applied for the NMR study of low-complexity regions of Htt exon1 combining cell-free expression using transcription-translation systems of *Escherichia coli* extracts and nonsense suppression for the site-specific isotopic labeling (Morató et al., 2020).

### 3.1 Understanding the Role of Specific Posttranslational Modifications in Amyloidogenesis Using Native Chemical Ligation

The combination of solid phase peptide synthesis (SPPS) with native chemical ligation (NCL) strategies provide the most efficient way to quantitatively introduce site-specific PTMs, and/or chemical/fluorescent labels or UAAs, into a protein of interest. Of note, amyloid-forming peptides/proteins, as exemplified by Aβ and IAAP, exhibit an intrinsic tendency to aggregation during SPPS and purification. Several strategies of chemical synthesis were implemented to prevent aggregation into β-sheet structures and improve solubility of difficult sequences (Butterfield et al., 2012), but these considerations are beyond the scope of this review. NCL, initially developed by Kent and co-workers, uses chemoselective reactions between the α-carboxyl and the α-amino groups of two unprotected peptides to form a native peptide bond (Muir and Kent, 1993; Dawson et al., 1994; Hackenberger and Schwarzer, 2008; Agouridas et al., 2019; Moon et al., 2022). This strategy allows introducing selective and quantitative modifications of amino acids without altering any usual peptide bond. However, the requirement of SPPS is limiting the length of affordable peptides to 50–60 residues, and multiple rounds of NCL can extend the polypeptide length at the expense of a significant reduction of the overall yield. To circumvent this issue, expressed protein ligation (EPL) implements the two-step reaction of NCL to generate semisynthetic proteins.

**FIGURE 3:**
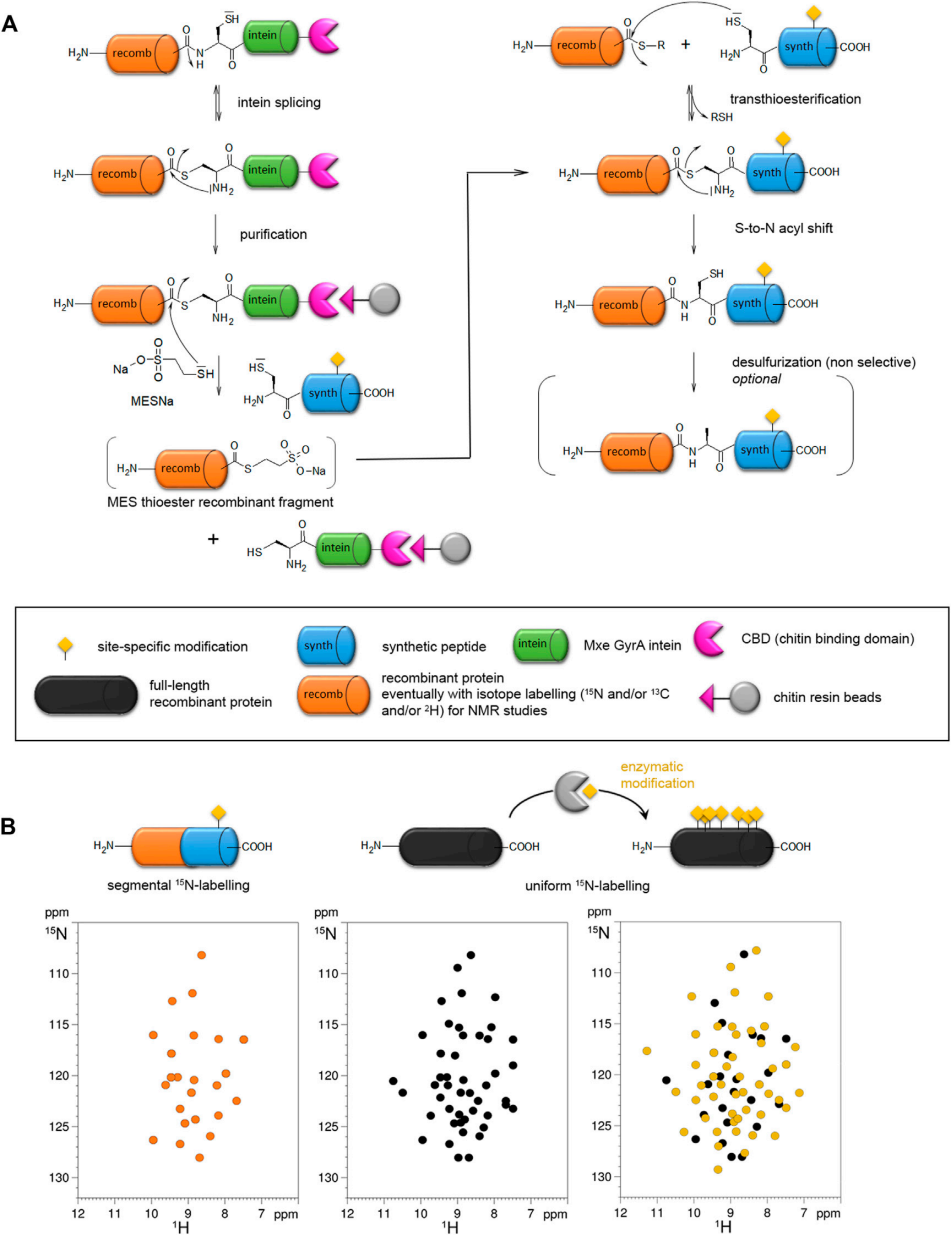
Expressed protein ligation (EPL) strategy for site-specific modification of proteins **(A)** and segmental isotope labeling for NMR studies **(B)**. **(A)** The synthetic peptide (blue) incorporating a site-specific modification (yellow) is obtained by SPPS and ligated to a recombinant protein fragment (orange) expressed in a heterologous system which can eventually be isotopically labeled for NMR study. The recombinant protein can be expressed as intein fusion protein (intein in green) with a CBD tag (pink) for purification on chitin beads. The reaction with sodium 2-mercaptoethane sulfonate (MESNa) and a synthetic peptide with a N-terminal cysteine leads to a semisynthetic protein. **(B)** In the NMR ^1^H-^15^N HSQC spectrum, only the ^15^N-labeled region of the protein is visible, therefore the synthetic region bearing the modification is invisible (orange spectrum). This strategy called segmental isotopic labeling allows a reduction of NMR signals in the spectrum relative to the full-length, uniformly labeled protein (black) without PTM (black spectrum) or with enzymatically installed PTMs (yellow spectrum). It is noteworthy that modifications of protein resonances observed locally for the modified residues and its neighbors in the primary sequence in the uniformly labeled protein cannot be observed in the semisynthetic protein with segmental labeling due to the absence of isotopic labeling in the region of PTM. However, this modification may have a long-range structural impact on residues of the ^15^N-labeled fragment due to conformational proximity that can be detected through perturbations of signals in the isotopically labeled region.

The first step of NCL consists of a transthioesterification, or reversible thiol/thioester reaction, by the nucleophilic attack of a N-terminal cysteine (through the side chain thiol function) of a synthetic peptide on the activated C-terminal thioester of another peptide. Both fragments will constitute the C-terminal and N-terminal parts of the full-length synthetic protein, respectively. The second step is a spontaneous and irreversible rearrangement called “S-to-N acyl shift” that restores a native peptide bond with a cysteine residue at the junction of both fragments (Figure 3A). Alternatively, EPL makes use of engineered mini-inteins as fusion to the expressed recombinant protein fragment of interest to introduce a C-terminal α-thioester. Inteins are self-processing domains involved in posttranslational protein splicing processes. The intramolecular rearrangement at the intein N-terminal cysteine generates the C-terminal α-thioester of the expressed fragment. The functionalized fragment can be next ligated to a synthetic fragment containing a N-terminal cysteine to generate a semisynthetic protein (Figure 3A). As the C-terminal fragment is obtained by SPPS, any modification (PTM, probe, other chemical modification of amino acid) can be easily inserted into a specific position of this region of the final semisynthetic protein. A synthetic N-terminal fragment bearing a thioester can also be ligated to an expressed C-terminal fragment. In this case, a N-terminal cleavable fusion tag, e. g. His_6_-SUMO tag, is used to afford the N-terminal cysteine required for the subsequent ligation step (Chiki et al., 2021). The use of a SUMO tag offers the advantages of improving protein expression and solubility, and facilitates protein handling and purification. All these strategies may require final steps of refolding, oxidation of the ligation product, and eventually desulfurization of the cysteine residue at the ligation site to restore a native alanine residue. They are better suited to introduce modifications of the terminal regions of semisynthetic proteins. Introducing modifications in the central region requires two ligation steps with a three-segment strategy (or more) at the expense of the reaction yield. We refer the readers to references (Hackenberger and Schwarzer, 2008; Agouridas et al., 2019) for extensive, general considerations in addressing NCL/EPL including the choice of ligation site, protection/deprotection strategies, desulfurization reactions.

The NCL strategy has been successfully used to introduce UAAs bearing chemical modifications (Chuh et al., 2016), e.g. metabolically stable phosphonate and difluoro-phosphonate analogs of phosphorylated residues. It proved to be also useful for segmental isotopic labeling of large proteins for structural analyses by NMR spectroscopy (Vogl et al., 2021). In this field, EPL helped to partially overcome the size limitations inherent to solution-state NMR by alleviating the number of resonances and thus, spectral overlap (Figure 3B). Additionally, NMR offers an orthogonal viewpoint to the study of PTMs from an analytical, mechanistical, structural and functional perspective (Theillet et al., 2012). By providing homogenous PTM patterns and reducing the number of PTM sites, EPL can help characterizing PTM-driven conformational and functional changes, although with a limited knowledge on the local conformational impact due to the absence of isotopic labeling of the synthetic fragment containing site-specific PTM(s) (Figure 3B). The occasional use of isotopically labeled amino acid synthons for SPPS may partially overcome this limitation albeit with a significant cost increase. An alternative route to PTM incorporation is the chemoenzymatic semisynthesis employing *in vitro* enzymatic modification of an expressed fragment, or co-expression of the fragment with the modification enzyme in *E. coli*, preceding the ligation step (Pan et al., 2020; Chiki et al., 2021; Kolla et al., 2021; Pan et al., 2021). This approach is limited to either small or mutated fragments, or scarce PTMs (such as pTyr) to reduce the number of modification sites or alternatively, it requires an efficient purification method to isolate a homogenously modified fragment preferably before ligation.

As exemplified by the whole proteome, phosphorylation is an important PTM in neurodegenerative diseases through modifications of most, if not all, amyloid proteins (Tenreiro et al., 2014). Involved in the crosstalk with phosphorylation, *O*-GlcNAcylation has been extensively investigated by semisynthetic approaches since the low enzymatic activity of OGT *in vitro* still limits the purely enzymatic strategy (Schwagerus et al., 2016; Balana and Pratt, 2021). Among other PTMs commonly found in amyloid proteins, lysine modifications such as acetylation, methylation (poly)ubiquination and SUMOylation are also explored by EPL. Several chemical biology groups have thus addressed the role of site-specific PTMs of amyloid proteins by developing various strategies of chemical synthesis, EPL and chemoenzymatic semisynthesis to improve the efficiency and yield of the ligation reaction as well as traceless purification of the ligation product (Ansaloni et al., 2014; Reimann et al., 2015; Levine et al., 2019; Kolla et al., 2021). Because, the NCL/EPL strategy depends on protein sequence and length, and on the nature and site(s) of PTM, specific strategies are elaborated in a case-by-case basis, i.e. for a specific amyloid protein and PTM. In the following, some examples are given to illustrate both the NCL/EPL approach involved and the findings related to the amyloidogenic pathways.

#### 3.1.1 α-synuclein

Either a three-fragment ligation or chemoenzymatic semisynthesis approach was employed to achieve tyrosine phosphorylation of α-synuclein at Y39 to evaluate its impact on aggregation properties and toxicity (Pan et al., 2021; Pan et al., 2020; Dikiy et al., 2016). Phosphorylation of Y39 was made possible by the chemoenzymatic strategy because the three other Tyr residues of α-synuclein are all located in the C-terminus that was recombinantly expressed in bacteria while the N-terminal part was enzymatically phosphorylated before ligation. Furthermore, desulfurization following the ligation steps restores native residues at junction sites considering the absence of cysteine in the native sequence of α-synuclein. It is noteworthy that recombinant expression of all fragments further assembled by ligation allowed the uniform ^15^N isotopic labeling of the site-specific pY39 α-synuclein for NMR studies (Vogl et al., 2021). It has been shown using these strategies that pY39 accelerates aggregation kinetics (Pan et al., 2020; Dikiy et al., 2016) and alters fibril morphology of α-synuclein with pY39 attracting the entire N-terminus within the fibril core through an array of electrostatic interactions giving rise to the largest fibril core (residues 1–100) ever seen for the α-synuclein amyloid fibrils (Figure 2) (Zhao et al., 2020). The homogenously phosphorylated α-synuclein at S129, one of the major pathological marks of Lewy bodies in PD, forms a different amyloid fold and has different propagation properties related to the wild-type protein, indicating the formation of a distinct strain associated to higher toxicity (Ma et al., 2016). Be it either localized at pY39 or pS129, single phosphorylation of α-synuclein provided by NCL highlights a capacity of site-specific phosphorylation to modulate the fold of the amyloid structure and pathological strain properties. In contrast, phosphorylation at Y125 does not alter the structure and morphology of the α-synuclein fibrils (Hejjaoui et al., 2012).

As α-synuclein has multiple *O*-GlcNAcylation sites, the site-specific *O*-GlcNAcylation provided by EPL underscored its inhibitory role in fibril assembly on either T72, T75, T81 or the three together, or S87, with the strongest effect observed for T75, all these sites being embedded within the fibril core (Marotta et al., 2015; Lewis et al., 2017; Li et al., 2018b; Zhang et al., 2019b; Levine et al., 2019; Tavassoly et al., 2021). Additionally, the combination of three T72/T75/T81 GlcNAc *O*-glycosylation reduces the aggregation of wild-type α-synuclein or aggregation-prone mutant (A53T) that causes early-onset, familial PD. *O*-GlcNAcylation alters the structure of aggregates in a site-specific manner, reduces the cytotoxicity of extracellular fibrils and impaired the calpain-mediated proteolysis of α-synuclein. Interestingly, the Pratt’s group has highlighted that *O*-GlcNAc at T72 is unique in its aggregation inhibitory properties as compared to other single sugars including *O*-GalNAc suggesting an effect that extend beyond its mere bulky size and polar properties, owing likely to the chirality of specific asymmetric carbons that would require further investigations (Galesic et al., 2021).

**FIGURE 4:**
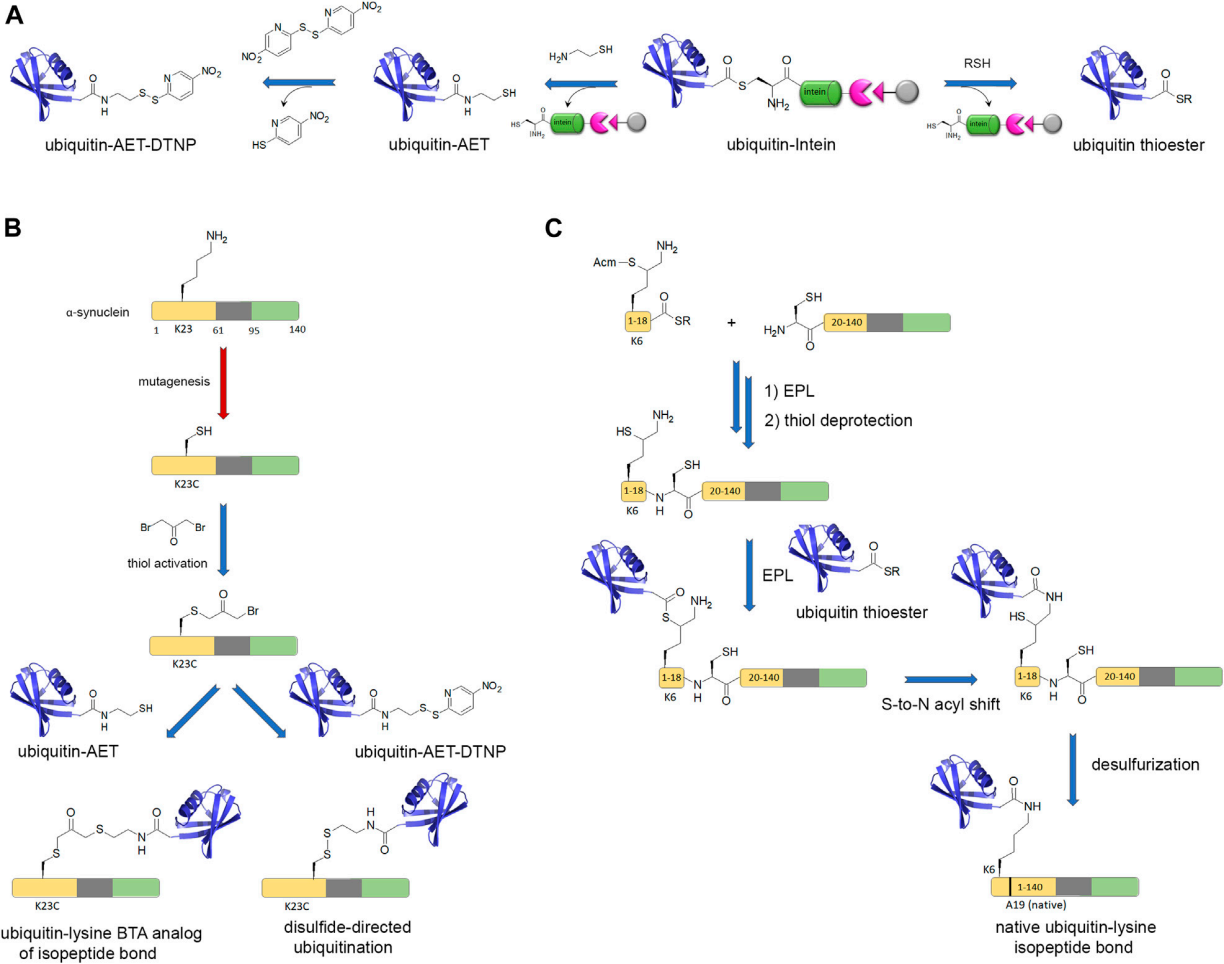
Chemical biology strategies of protein ubiquitination. **(A)** Posttranslational chemical mutagenesis and EPL approaches involve both the preparation of a C-terminal activated ubiquitin from a ubiquitin-intein fusion protein that provides a reactive C-terminal thioester. **(B)** Posttranslational chemical mutagenesis exemplified for K23 ubiquitination of α-synuclein requires first the thiol activation of a cysteine residue obtained by site-directed mutagenesis at the PTM site. The activated thiol is further involved in a reaction with ubiquitin-aminoethanethiol (ubiquitin-AET) leading to a bis-thio-acetone (BTA) analog of the ubiquitin-lysine isopeptide bond (Lewis et al., 2016). Alternatively, a ubiquitin-AET activated by 2,2′-dithiobis(5-nitropyridine) (ubiquitin-AET-DTNP) can react with the cysteine thiol in a disulfide-directed ubiquitination (Moon et al., 2020). **(C)** The EPL strategy involves first, the synthesis of full-length α-synuclein by EPL of two fragments, a N-terminal peptide (fragment 1–18) bearing a protected δ-mercaptolysine at position K6 for further ubiquitin linkage and a C-terminal recombinantly expressed fragment with a N-terminal cysteine. After NCL and thiol deprotection of the δ-mercaptolysine, EPL with the recombinantly expressed ubiquitin with C-terminal thioester and subsequent desulfurization provide the full-length, native α-synuclein with a native ubiquitin-lysine isopeptide bond (Hejjaoui et al., 2011). Chemical reactions are indicated by blue arrows and enzymatic reactions by red arrows.

Finally, the investigation of site-specific ubiquitination of α-synuclein involved a strategy of disulfide-directed ubiquitination, as implemented by Pratt and co-workers, requiring a lysine-to-cysteine point mutation for installation of ubiquitin through a disulfide bond instead of the native isopeptide bond. This strategy involved the recombinant production of a ubiquitin-intein fusion protein followed by reaction with cysteamine that simultaneously affords intein cleavage and thiol functionalization of ubiquitin C-terminus (Figures 4A,B). The subsequent thiol activation as a disulfide or a redox non-labile function by reaction with dibromoacetone provided ubiquitinated α-synuclein by reaction with a free thiol function positioned at diverse biologically relevant sites (K6, K10, K12, K21, K23, K32, K34, K46, and K96) (Meier et al., 2012; Abeywardana et al., 2013; Moon et al., 2020; Lewis et al., 2016). Whatever the ubiquitination or polyubiquitination site, an inhibition of α-synuclein aggregation was observed (Meier et al., 2012; Haj-Yahya et al., 2013). However, the disulfide bond is not chemically stable and other approaches were used to establish stable isopeptide linkage of ubiquitin. An alternative strategy formerly developed by Lashuel and co-workers although less flexible affords a native isopeptide linkage between K6 of α-synuclein and ubiquitin (Figures 4A,C). The synthesis of a thiol protected δ-mercaptolysine derivative is used in SPPS of a N-terminal fragment, then EPL with a recombinant C-terminal fragment provides the modified K6 within full-length α-synuclein. Subsequent NCL between the thiol function of δ-mercaptolysine and ubiquitin C-terminal thioester forms a native isopeptide bond between ubiquitin and the targeted lysine after subsequent desulfurization of the thiol handle of δ-mercaptolysine (Hejjaoui et al., 2011).

#### 3.1.2 Aβ Peptide

Aβ peptides from AD brain contain a variety of peptide lengths and post-translationally modified forms with phosphorylation, isomerization, and pyroglutamate, that modulate aggregation and propagation properties, and toxicity. In AD, modifications of the N-terminus in several Aβ subtypes were shown to both accelerate fibrillation and stabilize fibril structures. Phosphorylation of S8 is a modification of Aβ featured in late-stage of AD in the dispersed, membrane- and plaque-associated fractions (Kumar et al., 2011; Rijal Upadhaya et al., 2014). Homogenous phosphorylation of S8 of Aβ(1–40) peptide, afforded by SPPS, induces a higher cross-seeding efficiency when compared to unmodified Aβ(1–40) and a modification of fibril structure shown by ssNMR that highlights a tight N-terminus association with the amyloid core (Hu et al., 2019). In contrast, selective enzymatic modification of S26 stabilizes oligomeric forms but inhibits aggregation. Hence, it has been proposed that an ordered N-terminal region may be favorable to pS8-Aβ(1–40) strain to propagate to multiple Aβ subtypes by exerting a dominant role in fibril morphology. Furthermore, ordered N-terminal conformations in Aβ fibril structures may be biologically relevant as illustrated by brain-extracted Aβ40 and Aβ42 fibril structures. In contrast, Y10 *O*-glycosylation of Aβ with a (Galβ1-3GalNAc) disaccharide or sialylated trisaccharide (NeuAcα2-3Galβ1-3GalNAc) was shown to destabilize the amyloid structure to form a new fibril polymorph with a less stable protofilament interface rendering fibrils more prone to degradation in agreement with short tyrosine *O*-glycosylated forms found in CSF of AD patients (Liu et al., 2021).

Recently, the O-GlcNAc glycosylation of small heat shock proteins (sHSPs) by EPL in the Pratt’s group has extended targeting modulators of amyloid aggregation to site-specific PTMs of their cofactors. O-GlcNAc modification was found to selectively improve the anti-amyloid activity of HSP27 and other sHSPs in aggregation of both α-synuclein and Aβ(1–42), by competing with intramolecular interactions. The subsequent conformational rearrangement upon HSP27 O-GlcNAcylation most likely favors the formation of larger multimers with increased activity (Balana et al., 2021). Besides highlighting a protective role of O-GlcNAc in amyloid aggregation, this work opens new avenues for orthogonal strategies to inhibit the formation of amyloid fibril by targeting their cofactors.

#### 3.1.3 Tau Protein

**FIGURE 5:**
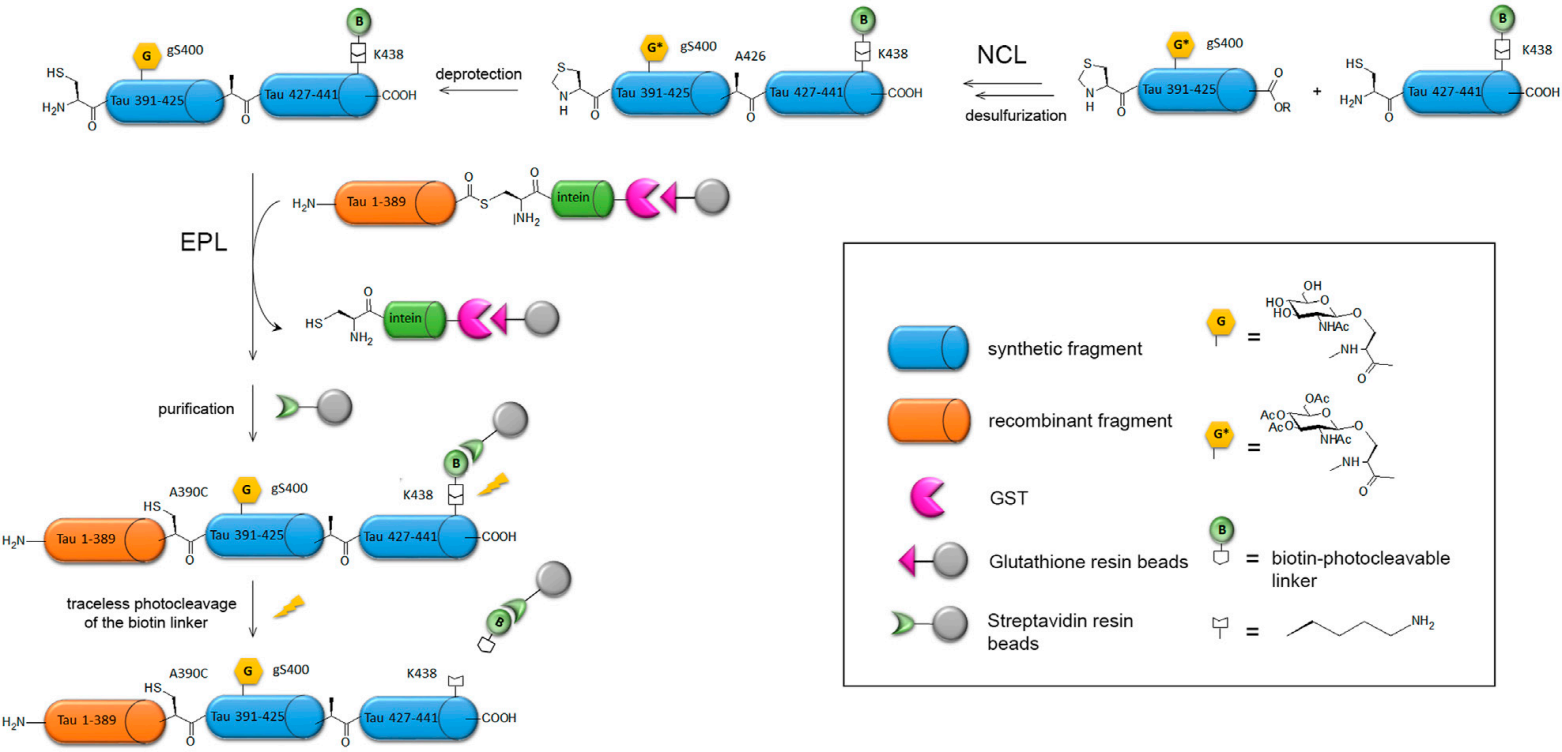
EPL strategy applied to the semisynthesis of full-length tau-S400-O-GlcNAc protein (Schwagerus et al., 2016). The C-terminal synthetic fragment incorporating the S400-O-GlcNAc modification and a biotin selectively linked to K438 residue for purification is obtained by NCL of two synthetic peptides, followed by desulfurization to restore the native A426 at the ligation site and deprotection of the GlcNAc moiety. The resulting C-terminal S400-O-GlcNAc peptide is then ligated by EPL with the N-terminal fragment recombinantly expressed as intein-GST fusion purified on glutathione affinity chromatography. The ligation product is isolated from the unreacted recombinant fragment through streptavidin-biotin affinity chromatography and traceless released by photocleavage of the biotin linker. The final product is the full-length protein tau-S400-O-GlcNAc with a A390C mutation at the EPL ligation site that cannot be recovered by desulfurization due to the presence of two native cysteine in tau sequence.

The long sequence of tau protein combined to a high proportion of polar residues (Ser, Thr, Lys, Arg) and Pro in the proline-rich domain coincides with a large number of PTMs such as phosphorylation, acetylation, ubiquitination, SUMOylation, methylation and *O*-GlcNAcylation. Hyperphosphorylation was shown to have a dominant role in tau fibril formation and loss-of-function in MT stabilization (Alonso et al., 1994; Morishima-Kawashima et al., 1995b; Alonso et al., 2001; Tepper et al., 2014), but other PTMs may have important effects in modulating tau functions and amyloid assembly. Site-specific PTMs may exert antagonist roles as well with some phosphorylation sites promoting aggregation while others have an inhibitory effect (Schneider et al., 1999; Liu et al., 2007; Despres et al., 2017; Brotzakis et al., 2021). Tuning on and off specific phospho-epitopes was shown to modulate tau function on tubulin polymerization or the Pin1-mediated regulation of tau dephosphorylation by PP2A (Amniai et al., 2009; Landrieu et al., 2011). The use of enzymes for introducing a limited pool of PTMs generally requires multiple mutations into an amino acid that cannot be modified as exemplified by the restriction of phosphorylation patterns to single phospho-epitopes, such as AT8 and PHF-1, for functional investigations (Amniai et al., 2009; Landrieu et al., 2011; Despres et al., 2017; Cantrelle et al., 2021). Alternatively, mimetics of PTM-amino acid may be inserted at single or multiple place mimicking a permanently modified state (Eidenmüller et al., 2001; Min et al., 2015). First attempts of full-length tau EPL semisynthesis were made by Hackenberger and co-workers, for the introduction of phosphorylation on S404 in the C-terminal region by ligation of a C-terminal phosphorylated peptide (from residue 390–441) to a N-terminal recombinantly expressed fragment (from residue 1–389) fused to intein (Broncel et al., 2012). Further improvement of the ligation strategy was required to afford the triple phosphorylation of PHF-1 epitope (pS396/pS400/pS404) or S400 O-GlcNAcylation owing to the length of the synthetic peptide (52 residues) (Reimann et al., 2015; Schwagerus et al., 2016). Hence, the synthesis of the C-terminal peptide involved NCL of two fragments followed by desulfurization, then EPL with the recombinant N-terminal fragment provides the full-length protein. A traceless purification strategy was also implemented through the use of a photocleavable biotin handle to purify the ligation product (Figure 5).

The presence of two native cysteine in tau primary sequence prevents from desulfurization of the EPL product. To circumvent this issue, the Lashuel’s group has exploited the native cysteine residues (C291 and C322) in a three-fragment ligation strategy for the synthesis of the tau K18 fragment (residues 243–372) corresponding to the microtubule-binding repeats (MTBR) containing four repeat/inter-repeat sequences, a region overlapping the core of amyloid fibrils from diverse tauopathies. This approach involves the native cysteine at the NCL junction sites preventing any introduction of non-native cysteine. The total chemical synthesis of K18 allowed the installation of phosphorylation at single (pS356) or multiple sites (pS262/pS356 and pS258/pS262/pS356) known to modulate tau function in microtubule polymerization/binding and amyloid assembly (Schneider et al., 1999). The site-specific phosphorylation of K18 proved to inhibit its aggregation *in vitro*, its seeding property in biosensor cells, and its ability to promote microtubule polymerization (Haj ‐ Yahya et al., 2020). The same strategy including first NCL of a C-terminal synthetic fragment was used in combination with an expressed N-terminal tau fragment (residues 2–245) for EPL allowing the semisynthesis of full-length tau after ligation of five fragments with either pY310, Ac-K280 or pS396/pS404 modification (Haj-Yahya and Lashuel, 2018). To obtain wild-type tau, two desulfurization steps were performed restoring native alanine at ligation sites before the incorporation of both fragments bearing native cysteine residues. The site-specific acetylation of K280 reproduces with increased outcome the effect of K280Q acetylation mimicking mutant on tau aggregation by forming rather oligomers and short fibrils instead of the long filaments observed for unmodified tau consistently with the increased seeding efficiency and toxicity of this mutant in AD models of tau pathology (Min et al., 2010; Cohen et al., 2011; Gorsky et al., 2016).

PHF-tau isolated from AD brains is conjugated to ubiquitin at multiple lysine residues within the MTBR, such as monoubiquitination at K254, K257, K311 and K317, and K48-linked polyubiquitination at K254, K311 and K353. Hence, the chemoselective disulfide coupling reaction implemented for ubiquitination of α-synuclein was similarly employed for the site-specific monoubiquitination of the K18 tau fragment at K254, K311 or K353 in which both native cysteine were mutated into alanine to prevent unwanted conjugation of ubiquitin. In contrast to multiple enzyme-derived ubiquitination that prevents the formation of both prefibrillar oligomers and amyloid fibrils, the semisynthetic ubiquitinated tau conjugates form oligomers and filaments to different extent with ubiquitination at K311 position being the strongest inhibitor (Munari et al., 2020).

#### 3.1.4 Huntingtin

Mutant Huntingtin as a primary cause of Huntington’s disease is characterized by an abnormal expansion of polyglutamine (poly Q) tract at the N terminus which has been described as pathological when exceeding 40 glutamine repeats instead of the normal 9 to 35. The length of the expanded polyQ tract was suggested to be proportional to disease severity. The first 17 N-terminal residues of Htt act in modulating Htt structure, interactions and aggregation. Hence, polyQ expansion was shown to cause misfolding of the N-terminus and could play a key role in aggregation and toxicity. The polyQ expansion in mutant Htt exon1 causes conformational changes and increases phosphorylation at multiple sites across the entire protein (Jung et al., 2020). It was suggested that phosphorylation of Htt N-terminus could reverse the conformational rigidity related to polyQ expansion and improve the toxic properties. Phosphorylation of the N-terminal fragment of Htt at T3 homogeneously obtained by EPL, either in wild-type (23Q) or mutant Htt protein (43Q) was shown to stabilize the α-helical conformation of the N-terminal 17 amino acids and significantly inhibit aggregation while K6 acetylation reverses the inhibitory effect of pT3 without exhibiting any intrinsic inhibitory effect by itself (Ansaloni et al., 2014; Chiki et al., 2017). More recently, a new chemoenzymatic semisynthesis of Htt N-terminal fragment (1–170) that forms nuclear and cytoplasmic inclusions in cell and animal models of HD enables the introduction of phosphorylation in exon3 (Kolla et al., 2021). Phosphorylation of T107 was shown to have opposite roles on Htt(1–171)-43Q aggregation depending on the phosphorylation status of S116 highlighting the benefits of site-specific PTM installation through semisynthesis in deciphering the PTM code that regulates the aggregation properties of amyloidogenic proteins.

#### 3.1.5 TDP-43

The semisynthesis of TDP-43 prion-like domain bearing a site-specific phosphorylation at S404 required the use of denaturing conditions to solubilize protein fragments expressed in bacterial expression systems. Indeed, the strategy of EPL through purification of full-length TDP-43 or prion-like domain as protein thioesters by the traditional fusion intein-CBD was unsuccessful to produce soluble TDP-43 variants in contrast to α-synuclein, Htt or tau proteins. Hence, the selected approach made use of a polyhistidine tag attached to a C-terminal cysteine residue which is submitted to S-cyanylated cysteine-promoted C-terminal hydrazinolysis to remove the tag. The tag removal then leaves a reactive C-terminal hydrazide that can be converted into a thioester for further NCL with the C-terminal pS404 fragment afforded by SPPS. Using this procedure, phosphorylation of S404 of TDP-43 prion-like domain was shown to accelerate the amyloid aggregation of the TDP-43 prion-like domain and worsen cytotoxicity (Li et al., 2020a).

Although the semisynthesis of post-translationally modified proteins has taken advantage of the NCL/EPL strategy, its major drawback is related to the need for a combined expertise in both peptide synthesis and chemical ligation reactions, and when applicable, the refolding of protein fragments of interest may be limiting. Expansion of the genetic code using amber stop codons and an orthogonal tRNA/tRNA synthase pair is an alternative strategy to introduce site-specific PTMs such as phosphoserine, acetyl-lysine, mono-/di-methyl-lysine, and UAAs (Wang et al., 2006; Tarrant and Cole, 2009; Park et al., 2011; Ge and Woo, 2021). However, this route has not been extensively applied so far to amyloid proteins. Finally, a more flexible method analogous to NCL implemented the sortase-mediated ligation (SML), or sortagging, which benefits from the transpeptidase activity of bacterial Sortase A (SrtA) enzymes that recognize with a high substrate specificity LPXT motifs and conjugate them to oligoglycine units through a native peptide bond (Dai et al., 2019; Li et al., 2020b). This versatile ligation approach demonstrates broad applications *in vivo* and *in vitro* for protein engineering, for instance by ligating a peptide substrate bearing PTMs, UAAs or other labeling probes (obtained from SPPS) into sortagging reactions. The directed evolution of SrtA to recognize the LMVGG sequence of Aβ (residues 34–38) enabled labeling of endogenous Aβ in human CSF for sensitive detection and conjugating a hydrophilic peptide to Aβ42 that blocks amyloid aggregation (Podracky et al., 2021). Dual-color fluorogenic aggregation sensors were introduced by SML to monitor protein co-aggregation in transthyretin amyloidosis by a thermal shift assay (Bai et al., 2021).

### 3.2 Posttranslational Chemical Mutagenesis

#### 3.2.1 Reactions With Cysteine Thiol Function

The site-specific chemical installation of PTMs made use of the reactivity of cysteine thiol group and requires first the site-directed mutagenesis of the PTM site to introduce a non-native cysteine residue, and eventually replacing native cysteine(s) by serine or alanine residues. An acetyl-lysine analog can be introduced through Lys-to-Cys mutation at a specific site and subsequent thiol alkylation with methylthiocarbonylaziridine leading to a thioether bond bearing a terminal S-methyl thiocarbamate group as acetyl mimic (Huang et al., 2010). Other chemical approaches for the site-specific ubiquitination of proteins, comprise the formation of thioether bonds (Hemantha et al., 2014) or triazole moiety through Copper(I)-catalyzed azide-alkyne cycloaddition (CuAAC) (Rösner et al., 2015) as stable analogs of the ubiquitin isopeptide bond. Mono- and di-methylated (symmetric or asymmetric) arginine-containing proteins can be prepared by reaction of cysteine thiol with *α*,*β*-unsaturated amidines leading to the corresponding methylated arginine analogs (Le et al., 2013). The chemical installation of mono-, di- and tri-methyl-lysine analogs can be provided by thiol alkylation with the respective N-methylated (2-chloroethyl)-ammonium halide (Simon et al., 2007). However, none of these reactions were applied so far to amyloid proteins to our knowledge.

#### 3.2.2 Versatile Chemistry of Dha/Dhb Precursors

**FIGURE 6:**
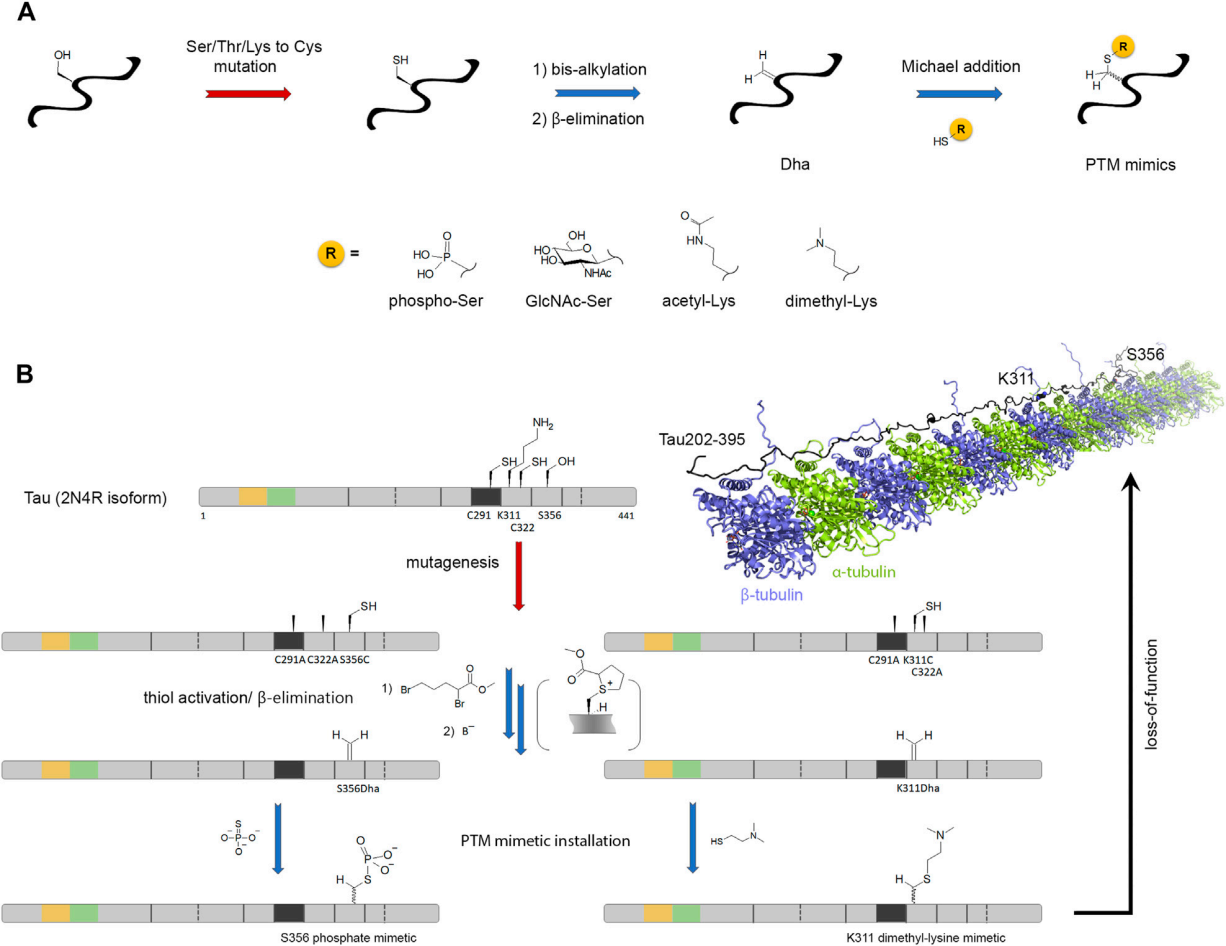
Posttranslational chemical mutagenesis. **(A)** General scheme of the posttranslational chemical mutagenesis approach for incorporation of PTM mimics by Michael addition of PTM-thiol derivatives on dehydroalanine (Dha) alkene function. The latter is obtained from serine/threonine/lysine-to-cysteine mutation at the PTM site and β-elimination of the bis-alkylated thiol function of cysteine. Addition of thiophosphate, S-GlcNAc, N-acetylcysteamine and captamine generates mimetics of phosphorylation, O-GlcNAcylation, N-acetylation and N,N-dimethylation, respectively. The thio-ether bond formation leads to a racemization of amino acid Cα. **(B)** This approach is illustrated by S356 phosphorylation and K311 acetylation in tau protein for the investigation of the PTM effect on loss-of-function in microtubule polymerization (PDB ID: 7PQP) (Brotzakis et al., 2021; Lindstedt et al., 2021). Chemical reactions are indicated by blue arrows and enzymatic reactions by red arrows.

Besides NCL, an increasing number of chemical methods has been developed to encode or decode PTMs in various proteins, as extensively described in excellent reviews (Chuh et al., 2016; Ge and Woo, 2021; Yang et al., 2018). A versatile approach is the chemical editing of amino acids into protein after their synthesis, thus independently of the ribosome or any enzymatic machinery. Dehydroalanine (Dha)/dehydrobutyrine (Dhb) are versatile chemical precursors to a range of PTMs including phosphorylation, glycosylation, methylation, acetylation, lipidation, and their analogs (Figure 6A). Dha/Dhb precursors can be introduced chemically or genetically into proteins (Jones, 2020; Yang et al., 2019). These reactive Ser/Thr analogs allow following olefin addition chemistry introducing a wide variety of PTMs in a strategy named “posttranslational chemical mutagenesis” as well as UAAs or molecular probes for purification or detection purposes. A simple way to obtain Dha site-specifically is through β-elimination of the cysteine thiol function that requires 1) the site-directed mutagenesis of the PTM target site to install a cysteine precursor and 2) the Cys-to-Dha conversion through a bis-alkylation elimination reaction (Figure 6A) (Chalker et al., 2011). Although the Cys-to-Dha conversion was used to map cysteine accessibility in a globular protein (Bertoldo et al., 2017), all cysteines are equally reactive in IDPs and, whenever needed, native cysteines must be mutated. An alternative strategy is the genetic introduction of Dha precursors reactive to β-elimination, like phosphoserine, through genetic code expansion using amber codon suppression (Yang et al., 2016).

Subsequent to Dha formation, the installation of PTMs or PTM analogs is provided by Michael addition (Figure 6A). Thiol derivative precursors of PTMs are the most convenient way to introduce PTM but the formation of the thio-ether bond induces Cα racemization (and also at Cβ in the case of Dhb derivatives) which seems not to be an issue in most amyloid case studies. This strategy was employed to introduce PTMs in full-length tau protein like pseudo-phosphorylation at pS262, pS356, pS199 by reaction with thiophosphate (to generate a phosphocysteine) and lysine K311 acetylation (K311-Ac) and dimethylation (K311-diMe) by reaction with N-acetylcysteamine and captamine (to generate thio-ether mimetics of PTM-lysine), respectively (Figure 6B) (Brotzakis et al., 2021; Lindstedt et al., 2021). While K311-diMe and K311-Ac have little effect on the formation of microtubules, pseudo-phosphorylation of pS262 and pS356 in the MTBR as well as pS199 in the proline-rich region were shown to inhibit the polymerization activity of tau confirming the role of pS262/pS356 that was previously investigated by *in vitro* kinase phosphorylation and highlighting a novel functional role for pS199 that is located far outside the MTBR, in the proline-rich domain (PRD). This is not surprising however, since other AD-specific phosphorylation epitopes embedded in the PRD were shown to modulate microtubule assembly (Amniai et al., 2009). Alternative elegant and versatile strategies to create C-C (sp3-sp3) bonds instead of unnatural carbon-heteroatom bonds were described, albeit not applied so far to amyloid proteins. They made use of mild, carbon-centered free radical chemistry that are compatible with Dha precursor reactivity and aqueous conditions suitable for protein chemical reactions. Most importantly, free radicals are unreactive with most of functionalities found in biomolecules. The reaction of Dha with a wide range of free radical precursor halides generates a stabilized Cα free radical that is further quenched after formation of the C-C bond allowing the installation of side chain chemical diversity (unnatural, fluorinated, PTM or isotopically labeled amino acids) at a specific position with a high site- and regio-selectivity (creation of Cβ-Cγ bonds), albeit with Cα racemization (Wright et al., 2016; Aguilar Troyano et al., 2021).

### 3.3 Focus on the O-GlcNAc exception: the chemical biology toolbox for the installation, detection and enrichment of O-GlcNAcylated amyloid proteins

The O-GlcNAc modification of proteins, and more particularly amyloid proteins, has required special attention. As an addition of single sugar on Ser/Thr, this PTM is closely comparable to phosphorylation. In addition, the O-GlcNAcome tightly overlaps the phosphoproteome. Thus, protein O-GlcNAcylation has long been occulted due to its close interplay with phosphorylation and a lack of appropriate detection/analytical methods (Thompson et al., 2018). For example, the poor immunogenicity of this small, neutral sugar prevents from raising selective antibodies, and only a few site-specific O-GlcNAc antibodies are available (e.g. S400-O-GlcNAc tau-directed antibody from Vocadlo’s lab (Yuzwa et al., 2011)). Another major limitation is related to the low sub-stoichiometry of the O-GlcNAc modification requiring the development of specific enrichment methods for O-GlcNAc profiling. Finally, the absence of consensus sequences emerging from the number of O-GlcNAc databases hamper the prediction of O-GlcNAc sites. Particularly intriguing is the mode of regulation of both OGT and OGA as the only two enzymes encoded in the human genome to implement the whole O-GlcNAc modifications of the O-GlcNAcome facing approximately 500 kinases. This suggests the recruitment of regulating sub-units for specific substrate targeting.

Biochemical and genetic manipulations of OGT and its substrates has provided several solutions to produce enzymatically O-GlcNAc modified proteins as exemplified by the co-expression of OGT and the protein of interest in *E. coli* given that bacteria do not have any equivalent of OGT protein but produce UDP-GlcNAc in sufficient amounts. This strategy was employed with tau (Yuzwa et al., 2011) and α-synuclein (Zhang et al., 2017b) to address the O-GlcNAc modification pattern of tau and the role of O-GlcNAc in tau and α-synuclein aggregation, respectively. The co-expression OGT/substrate can be optionally accompanied by 1) the co-expression of GlmM and GlmU enzymes that participate to the bacterial UDP-GlcNAc biosynthesis and therefore help protein O-GlcNAcylation by enhancing intracellular UDP-GlcNAc concentration (Gao et al., 2018), and 2) the treatment of bacterial cultures and extracts with PUGNAc, a potent glycosidase inhibitor, since endogenous NagZ glycosidase can hydrolyze O-GlcNAc in exogenous glycosylated proteins, hence reducing the O-GlcNAc modification level (Goodwin et al., 2013). This strategy together with the *in vitro* incorporation of O-GlcNAc through incubation of the protein substrate with UDP-GlcNAc and expressed OGT (from heterologous expression system) lead to heterogenous, and most importantly, low sub-stoichiometry modification in most cases. The lack of regulatory sub-units in artificial systems could in part explain the inefficiency of protein O-GlcNAcylation with recombinant OGT or bacterial co-expression systems.

**FIGURE 7:**
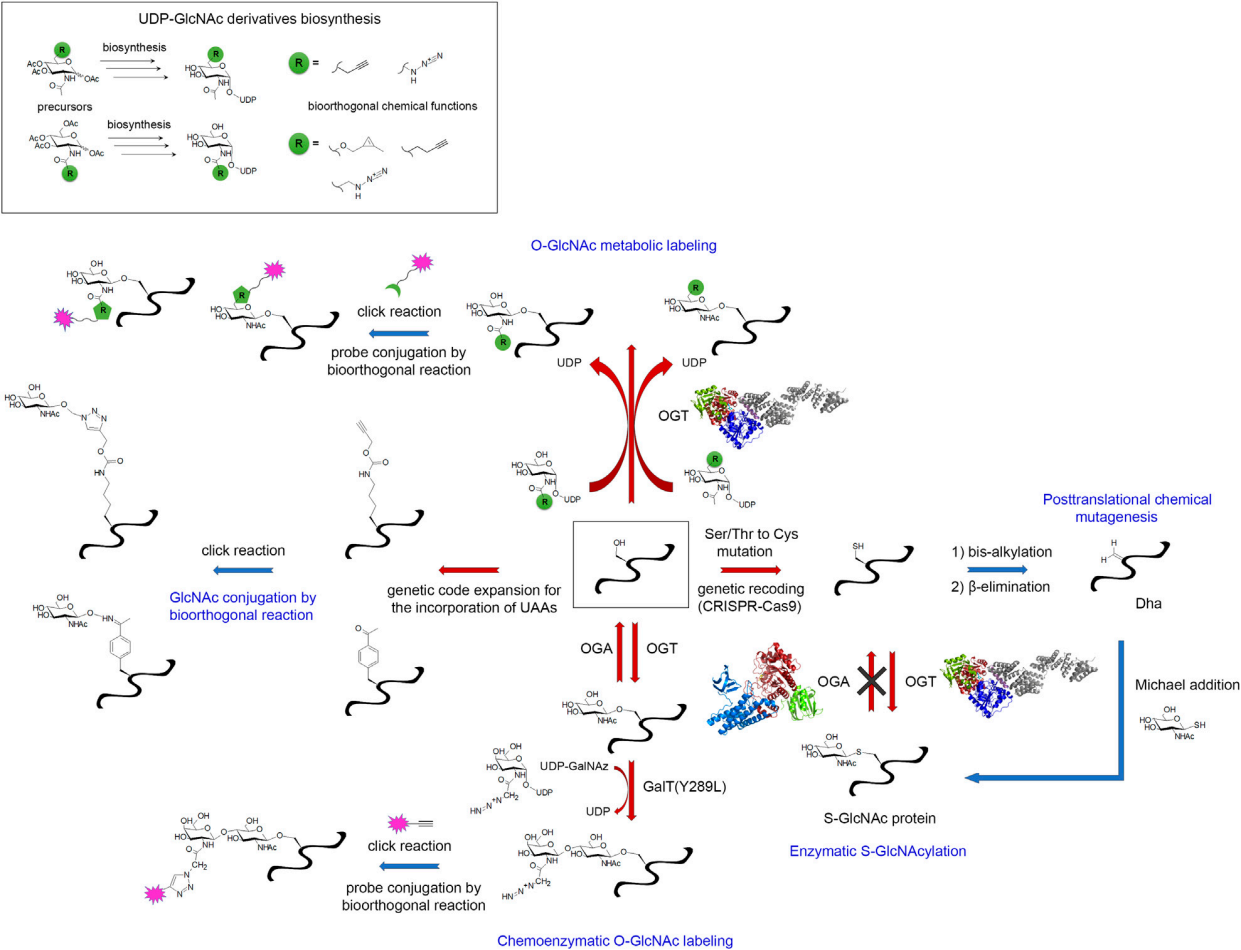
Posttranslational chemical mutagenesis, genetic code expansion and chemo-enzymatic reactions for incorporation of O-GlcNAc mimics and derivatives for O-GlcNAc detection/purification. Chemical reactions are indicated by blue arrows and enzymatic reactions by red arrows. Some examples of the manifold approaches for the incorporation of S-GlcNAc and O-GlcNAc derivatives are given. An extensive review of O-GlcNAc installation and detection can be found in (Saha et al., 2021). Site-specific O-GlcNAc mimics can be incorporated directly on the cysteine thiol function after Ser/Thr into Cys site-directed mutagenesis or using the gene-editing tool CRISPR-Cas9. This allows for instance the enzymatic installation of S-GlcNAc mimics with OGT leading to a stable GlcNAc derivative since hydrolysis by OGA is not possible. Alternatively, the engineered cysteine residue may also be an intermediate to provide a bioorthogonal reactive dehydroalanine (Dha) derivative after thiol bis-alkylation and subsequent β-elimination. The Dha handle is amenable to a Michael addition with a GlcNAc-thiol derivative for installation of a S-GlcNAc derivative. A third approach involved the incorporation of unnatural amino acids (UAAs) bearing a reactive handle amenable to “click” chemistry, e.g., acetophenone or propargyl-lysine, using the amber codon suppression strategy of genetic code expansion for the site-specific O-GlcNAc modifications. Coupling of the O-GlcNAc moiety is performed by azide-alkyne [3 + 2] cycloaddition. For detection or enrichment of O-GlcNAcylated proteins, labeling of O-GlcNAc is performed as a first step either (i) by “metabolic labeling” through oligosaccharide engineering that introduces chemical handles (azide, alkyne,…) directly on the GlcNAc group for subsequent reaction (via the biosynthesis of UDP-GlcNAc derivatives, depicted in the box) or (ii) by “chemoenzymatic labeling” through an enzymatic reaction of the O-GlcNAc group with a mutant of β1,4-galactosyltransferase (GalT-Y289L) that forms a glycosidic bond with a galactose derivative (GalNAz). In both strategies, the second step is the subsequent conjugation of reactive probes (e.g., fluorescent probes, mass tag, biotin…) for downstream enrichment or detection. The latter processes through a reaction of “click” chemistry making use of azide bioorthogonal reactivity through the copper(I)-catalyzed (CuAAC) or strain-promoted azide-alkyne cycloaddition (SPAAC).

The semisynthesis of α-synuclein incorporating a site-specific S-GlcNAcylation at S87C mutation site was used to probe the OGA activity on S-linked GlcNAc modification. Accordingly, the S-GlcNAc moiety was shown to be enzymatically stable upon OGA treatment*.* Moreover, it has the same effect than the O-linked counterpart in membrane binding of α-synuclein and inhibition of its fibrillar aggregation (De Leon et al., 2017). Following this approach, genetic methods implementing the CRISPR-Cas9 methodology for the site-specific Ser-to-Cys mutation of the glycosylation site has allowed incorporating S-GlcNAc derivatives with high level of protein modification in living cells (Gorelik et al., 2019) considering the ability of OGT to process cysteine residues while OGA cannot hydrolyze the S-linked GlcNAc sugar, thereby increasing the stability of the modification in the cell (Figure 7) (De Leon et al., 2017). Then, engineering of a hexosamine thioligase inspired from the GH20 hexoaminidase enzymatic mechanism and utilizing 4-nitrophenyl N-acetyl-β-d-glucosaminide (pNP-GlcNAc) as glycosyl donor has improved the GlcNAc S-linkage to targeted sites after Ser-to-Cys mutation (Tegl et al., 2019). The enzymatic S-GlcNAcylation of tau at S400C was performed using this approach. In addition, efforts were made to produce GlcNAcylated proteins both *in vitro* and *in vivo* through “proximity-induced reactions” improving levels and detection of GlcNAcylated proteins (see paragraph 4.3) (Ge and Woo, 2021).

Interestingly, O-GlcNAc seems to have an overall protective effect in amyloid aggregation of various proteins involved in neurodegenerative diseases, in part owing to its interplay with phosphorylation. The phosphorylation/O-GlcNAcylation crosstalk in tau protein derived from the overall modulation of O-GlcNAcylation (OGA inhibitors, mouse starvation) and distinct transgenic mouse models suffers from contradictory outcomes (Yuzwa et al., 2008; Yuzwa et al., 2012; Graham et al., 2014; Hastings et al., 2017) while *in vitro* studies highlight only a limited direct interplay between both PTMs at the protein level (Bourré et al., 2018; Cantrelle et al., 2021). Phosphorylation of S129 of α-synuclein, a marker of typical PD aggregates, was shown to be sensitive to the O-GlcNAc modulation, either by genetic or pharmacological upregulation of O-GlcNAcylation, while pS129 reduction has been observed in the homogenously T72-O-GlcNAc modified semisynthetic protein upon phosphorylation by different kinases (Marotta et al., 2015; Lee et al., 2020). Unraveling the regulation of tau or α-synuclein (hyper)phosphorylation and pathophysiological functions by the O-GlcNAc glycosylation would deserve further investigations with the targeted protein O-GlcNAcylation approaches.

The investigations of the functional impact of amyloid PTMs and PTM dysregulation in cell and *in vivo* systems require the isolation, detection and analytical characterization of posttranslationally modified proteins which proved to be particularly difficult in the general case of O-GlcNAcylated proteins. Indeed, both detection and/or enrichment of O-GlcNAc proteins usually apply succinylated wheat germ agglutinin (sWGA) lectin or pan-O-GlcNAc monoclonal antibodies, RL2 and CTD110.6, that detect O-GlcNAc proteins in cytosolic and nuclear extracts (Saha et al., 2021). However, sWGA although relatively selective has a low affinity for the single O-GlcNAc sugar while pan-O-GlcNAc antibodies have a low affinity and a limited specificity, sometimes exhibiting cross-reactivity with terminal GlcNAc of branched N-glycans (Isono, 2011; Tashima and Stanley, 2014). Significant advances using chemical biology tools during the last decade has strongly expanded the detection of O-GlcNAcylated proteins as a long-standing demand, and helped deciphering the functional role of O-GlcNAcylation as extensively reviewed in (Saha et al., 2021). Bioorthogonal chemical reactions have been implemented to afford a wide range of tools and probes for the detection or enrichment of O-GlcNAcylated proteins from *in vivo* systems (Figure 7). In particular, the O-GlcNAc enrichment methods combined with mass spectrometry-based proteomics have led to important advances in the profiling of protein O-GlcNAcylation in systemic approaches. As a first step, the labeling of O-GlcNAc is performed either by metabolic oligosaccharide engineering that consists of introducing several types of reporter groups (ketone, azide, alkene, alkyne, isonitrile) as chemical handles for subsequent reaction or, alternatively, by enzymatically coupling a galactose derivative using the specific reaction of β1,4-galactosyltransferase (GalT). The former approach uses the manipulation of the UDP-GlcNAc metabolic pathway and the OGT plasticity to a variety of UDP-GlcNAc derivatives (e.g. peracetylated N-azidoacetylglucosamine) to directly incorporate chemical reactive group in the GlcNAc moiety of glycosylated proteins (Moon et al., 2022; Saha et al., 2021). The latter approach achieved better efficiency by using a mutant of GalT (GalT-Y289L) tolerant to UDP-GalNAc derivatives, such UDP-GalNAz containing an azide group, as a substrate donor for glycosidic linkage to GlcNAc (Figure 7) (Khidekel et al., 2003). The second step is the subsequent reaction with diverse reactive probes (e.g. fluorescent probes, mass tag, biotin…) for downstream enrichment or detection. An example is the use of azide bioorthogonal reactivity through the [3 + 2] cycloaddition with alkynes. The development of a copper(I)-catalyzed azide-alkyne cycloaddition (CuAAC), termed “click” chemistry, improved the conditions of the initial Huisgen reaction to proceed in physiological conditions compatible with biomolecule functionalities. However, as Cu(I) is toxic in cellular environment, activated alkynes, e.g. induced by ring strain such as cyclooctyne, has been implemented in a strategy compatible with cellular environments named strain-promoted azide-alkyne cycloaddition (SPAAC) (Agard et al., 2004). The conjugation of fluorescent probes and biotin through a click chemistry reaction allowed subsequent O-GlcNAcylated protein detection and isolation while mass tagging (e.g. with resolvable polyethylene glycol mass tags) provides a rapid insight into the distribution of O-GlcNAc proteoforms.

## 4 Detecting and Characterizing Amyloids With Chemical Biology Tools

### 4.1 Chemical Tools for Amyloid Detection and Structural Characterization

#### 4.1.1 Small-Molecule Sensors of Amyloid Aggregation

**FIGURE 8:**
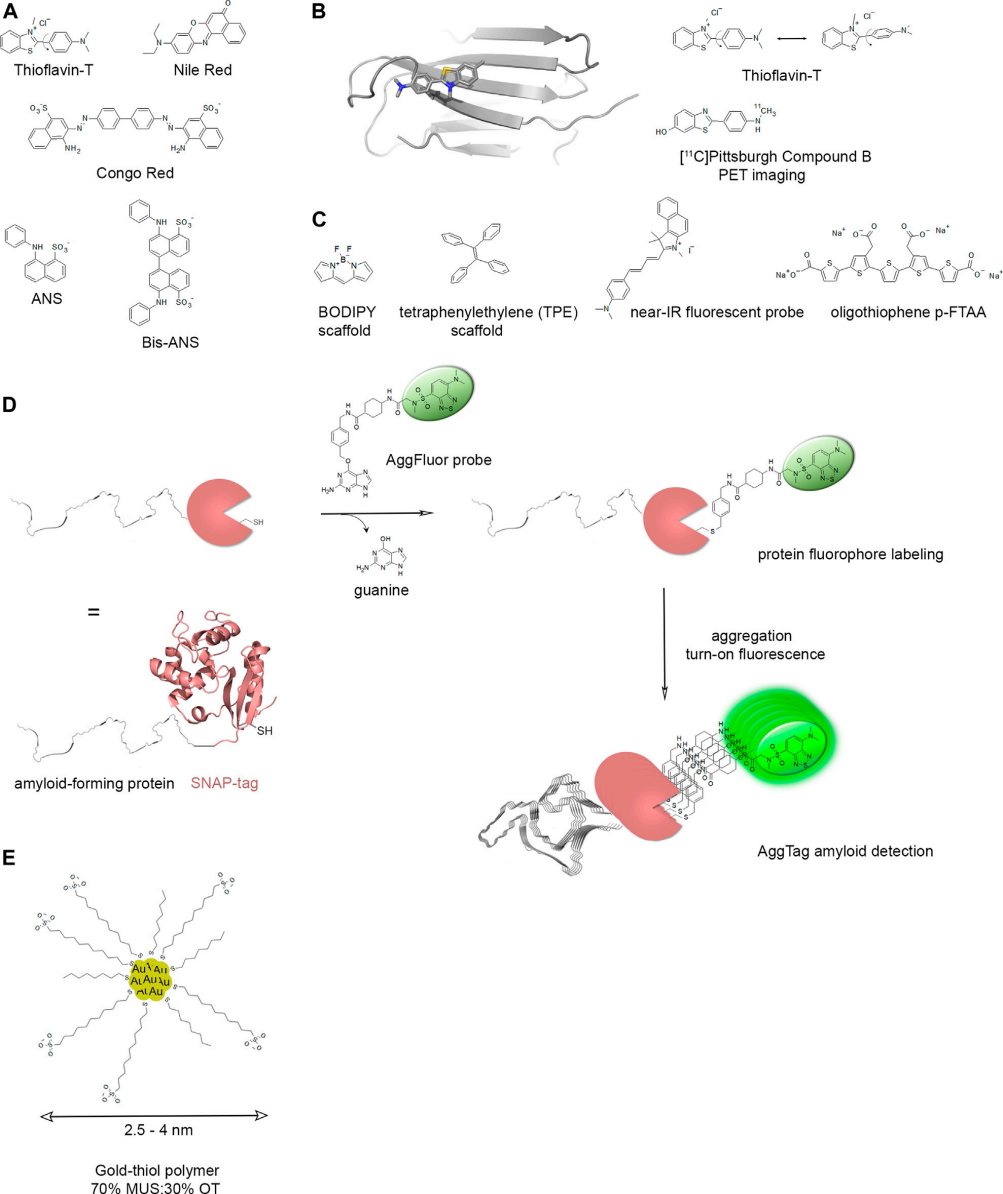
Detection of amyloids by fluorescence labeling **(A–D)** or functionalized gold nanoparticles **(E)**. **(A)** Structures of extrinsic fluorescent probes used for amyloid detection. **(B)** Aggregation-induced emission (AIE) molecules exemplified by Thioflavin-T (ThT) emit a fluorescent signal upon binding into hydrophobic pockets of amyloid β-sheet structures due to restriction of intramolecular rotation (PDB ID: 3MYZ). The structure of [^11^C]-Pittsburgh Compound B used for PET imaging is based on ThT structure. **(C)** Some examples of fluorescent molecules based on BODIPY or tetraphenylethylene (TPE) scaffolds are depicted as well as a near-infrared (near-IR) fluorescent probe and the oligothiophene p-FTAA (pentameric form of formyl thiophene acetic acid). **(D)** An example of fluorescent SNAP-tagging is depicted in a strategy called “AggTag” for amyloid detection. The engineering of a SNAP-tag amyloid protein allows coupling of an AggFluor probe through the selective reaction of a SNAP tag cysteine residue with O-benzylguanine derivatives. The subsequent aggregation of amyloid-forming proteins turns on fluorescence of the AggFluor probe. Various AggFluor probes has been designed to detect oligomers and amyloid fibrils, or amyloid fibrils selectively (Liu et al., 2018; Wolstenholme et al., 2020). **(E)** Functionalization of gold nanoparticles (gold-thiol polymer) with a mixture of 11-mercapto-1-undecanesulfonate: 1-octanethiol (MUS:OT) enables the selective detection of amyloid fibrils and polymorphism by cryo-EM imaging (Cendrowska et al., 2020).

Historical fluorescent probes for the detection of amyloids (e.g., ThS, ThT, ANS, Bis-ANS, Congo Red, Nile Red…) (Figure 8A) (Naiki et al., 1989; Biancalana and Koide, 2010; Krebs et al., 2005; Groenning, 2010; Amdursky et al., 2012) were recently complemented by a wide range of aggregation-induced emission (AIE) molecules (Tang et al., 2022; Tang et al., 2021) including hexaphenylsilole (HPS), tetraphenylethylene (TPE) and tetraphenylbutadiene that expand the detection of aggregates formed along the amyloidogenic pathway such as oligomers that are poorly detected by ThT, Congo Red and their derivatives. AIE molecules usually possess twisted structures in solution limiting the fluorescence yield by non-radiative transition (Kumar et al., 2017; Aliyan et al., 2019). Restricted intramolecular motions upon binding to amyloid β-sheets alleviates this phenomenon due to electrostatic and/or hydrophobic interactions, structural complementarity and/or chemical reactions (Figure 8B). For instance, increased fluorescence signal of new AIE probes with the size increase of Aβ aggregates enables a sensitive detection of Aβ aggregation and inhibition (Gour et al., 2019). Development of AIE probes includes the coverage of a large wavelength spectrum from the visible to the near-infrared (NIR) (Figure 8C). NIR probes are more convenient for super-resolution imaging of amyloids in cells and tissues by increasing the spatiotemporal resolution and photostability while reducing tissue damages. Probes for excited state intramolecular proton transfer (ESIPT) or a series of boron dipyrromethene (BODIPY) derivatives were designed with AIE properties for the follow-up of amyloid aggregation (Figure 8C) (Hu et al., 2011; Liu et al., 2019). A fluorescent BODIPY-based binuclear Zn(II) complex was rationally designed as a molecular probe with phospho-selective binding properties for peptides presenting phosphorylated groups at the relative *i* and *i*+4 positions. This molecule allowed for a selective detection of NFTs of hyperphosphorylated tau proteins in AD brain as it discriminated between NFTs and SPs of Aβ peptides in histological imaging of hippocampus sections from AD patients (Ojida et al., 2009). The luminescent amyloid-binding conjugated poly- and oligothiophenes (LCPs and LCOs) is a class of amyloid dyes of fibrillar aggregates for emission fluorescence staining or imaging (Figure 8C) (Åslund et al., 2009; Brelstaff et al., 2015; Klingstedt et al., 2011). LCOs are uniquely capable to discriminate different molecular architectures. The aggregate-specific emission is achieved due to conformational restriction of the thiophene backbone upon interaction with specific protein aggregates. Using the LCOs fluorescence signature, fibril polymorphism can be monitored both *in vivo* and *in vitro*. LCOs will be useful to link protein conformational features with disease phenotypes for a variety of neurodegenerative proteinopathies (Magnusson et al., 2014). Another series of molecular rotor fluorophores named AggFluor derived from the chromophore core of green fluorescent proteins (GFPs) was rationally designed with a gradient of viscosity sensitivity over a wide range (Figure 8D). AggFluor probes were capable of differentiating between soluble oligomers and insoluble aggregates through distinct turn-on fluorescence. This method was extended to dual-color imaging of aggregation and its modulation thereof with small-molecule proteostasis regulators in living cells (Wolstenholme et al., 2020).

#### 4.1.2 Functionalized Gold Nanoparticles

Probing of amyloids with gold nanoparticles or nanorods, or through immunogold labeling as gold nanoparticle conjugates to a specific antibody allowed mapping amyloid fibrils in tissue sections as well as identifying proteins and some of their molecular features. The presence of PTMs and cofactors, or the presence/accessibility of a particular sequence can be characterized in this manner (Fitzpatrick et al., 2017; Falcon et al., 2018b; Goedert et al., 1996; Al ‐ Hilaly et al., 2020). However, these methods do not provide detailed information on the fibril morphology and polymorphism owing to either the bulkiness of the particle itself or to the distance between the particles and coated fibrils due to the size of the antibody and/or nanoparticle attachment linker. Functionalization of gold nanoparticles of 3 nm-diameter with negatively charged ligands (e.g. 11-mercapto-1-undecanesulfonate: 1-octanethiol mixture) enables to detect amyloid polymorphism over a variety of amyloid sequences under hydrated conditions by cryo-EM imaging (Figure 8E) (Cendrowska et al., 2020). The functionalized particles act as contrast agents to rapidly stain fibrillar structures. Fibrils are stained either at the fibril edges as exemplified with Aβ40 and tau R2 peptides, or on the whole fibril surface as for proteins tau and Htt exon 1 with a 43 polyQ segment, probably through interactions with the fibril fuzzy coat. This nanoparticle labeling facilitates the characterization of fibril morphology thereby revealing distinct polymorphs. Moreover, differential fibril decoration also reflects distinct morphologies. The functionalized gold nanoparticles have highlighted a higher homogeneity of periodicity length distribution for *in vivo* related to synthetic fibrils in agreement with cryo-EM structures.

#### 4.1.3 Site-specific Introduction of Probes for the Study of Amyloids

Site-specific labeling of amyloid proteins allows to track conformation dynamics, interactions, modifications and seeding aspects by both *in vitro* approaches based on biophysical experiments and cell-based assays through microscopy imaging. Moreover, protein fluorescent labeling can be helpful in the screening of compounds for anti-aggregation or disaggregation activity by avoiding displacement of external fluorescent probes by competitive binding. The labels can be introduced with approaches similar to introduction of PTMs as detailed in paragraph 2 by incorporation of the labeled amino acid, or an amino acid amenable to bioorthogonal click chemistry (Arsić et al., 2022; Shimogawa and Petersson, 2021), by UAA mutagenesis, or based on suppression of stop codons. Another strategy of label incorporation is the conjugation of SPPS (peptide modified with a label) combined with NCL (see paragraph 2.1). In addition, the labels can be introduced using genetically engineered reactive tags for N- or C- terminal labeling, such as SNAP-tag (Keppler et al., 2003) CLIP-tag (Gautier et al., 2008) and Halo-tag (Liu et al., 2018) that react with derivatives of O-benzylguanine bearing a chemical probe to label the protein via a benzyl linker (Figure 8D). SNAP and CLIP or Halo tags can be simultaneously and differentially labeled with fluorescent reporters in living cells (Gautier et al., 2008; Jung et al., 2019). Compared with fluorescent proteins as GFP or mCherry, these tags are small and can be modified with more stable and brighter fluorophores.

The easiest way to introduce a tag is probably by using Cys residues as attachment points for maleimide or iodoacetamide derivatives, or other thiol reactive species (Gunnoo and Madder, 2016). The Cys residues can be native protein residues, or introduced at a unique position by site-specific mutagenesis, with replacement of the natural Cys residues by an unreactive amino acid. This has been conveniently used for α-synuclein labeling with an environment-sensitive fluorescent reporter to probe binding to lipid membranes and fibrillization by discriminating between unstructured, membrane‐bound and fibrillar states (Kucherak et al., 20182018). However, for the introduction of two different probes, a selective (de)protection of one of the two cysteine is required involving necessarily SPPS of a fragment at least of the protein of interest. Otherwise, a combination of different methods should be used. Cysteine conjugation was combined with amber codon suppression, transferase mediated N-terminal modification and NCL to produce α-synuclein variants bearing single or double site-specific fluorescent labels to probe conformational changes upon fibril formation and cellular uptake of fibrils (Haney et al., 2016; Haney et al., 2017).

Fluorescent probes differ whether investigating amyloidogenic processes *in vitro* or in living cells, and upon the fluorescence experiment employed being ensemble or single-molecule Förster resonance energy transfer (FRET), fluorescence polarization (FP), fluorescence correlated spectroscopy (FCS) or ESIPT. In any cases, careful considerations must be taken regarding the choice of labeling sites to avoid probe-induced perturbations of the protein conformation and fibrillization process even though small-molecule fluorophores are less invasive than their proteinaceous counterparts (e.g., GFP and its derivatives). Fluorophore are sensitive to change in their environments (e.g., exposure to the solvent, pH variation, ion concentration) as probed by FP and FCS. Introduction of two fluorophore labels gives access to FRET measurements based on distance-dependent fluorophore interactions. The distance between the fluorescent probes and dynamic fluctuation of these distances can be evaluated during fibril formation, upon interaction with small molecules (for example aggregation inhibitors) or due to a PTM. Single molecule FRET is of high interest when heterogeneity is considered, for example to characterize a population of oligomers. The use of fluorophore labels combined with microscopy imaging of live-cells is of importance to monitor cellular uptake, seeding and proteolysis.

**FIGURE 9:**
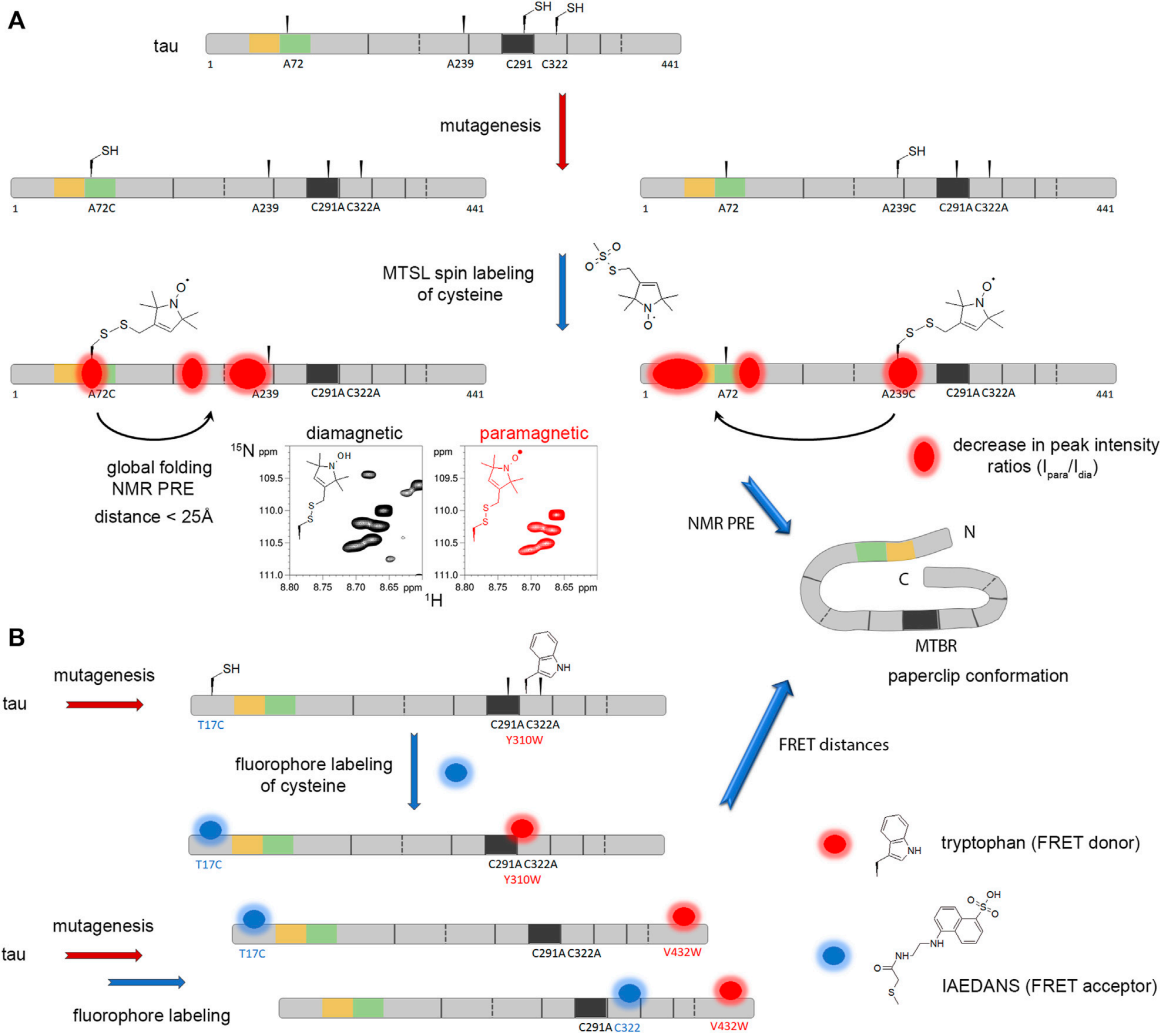
Nitroxide spin labeling and FRET fluorescent pair labeling for distance measurements by NMR, EPR and FRET exemplified in tau protein. **(A)** Nitroxide spin labels such as MTSL (S-(1-oxyl-2,2,5,5-tetramethyl-2,5-dihydro-1H-pyrrol-3-yl)methyl methanesulfonothioate) can be introduced on thiol function(s) of engineered cysteine residues obtained by site-directed mutagenesis for NMR experiments. Single spin label is used in paramagnetic relaxation enhancement (PRE) experiments. Double spin labeling of two distinct cysteine residues is used for paramagnetic relaxation interference (PRI) experiments (not shown in the figure). The same labeling strategy with single or double paramagnetic probes can be used in EPR experiments (not shown in the figure). Single spin labels are introduced at different positions along the protein sequence as illustrated by A72C and A239C labeling. Proximal and long-range perturbations of NMR signals (distance >10 Å up to 25 Å) from the spin label is indicated by red areas (Bibow et al., 2011). The PRE phenomenon is characterized by a selected decrease of peak intensities in the ^1^H-^15^N HSQC experiment acquired on the ^15^N-uniformly labeled protein and quantified by the I_para_/I_dia_ ratio corresponding to the intensities measured in the paramagnetic specie (I_para_) and the intensities measured in the diamagnetic specie (I_dia_) in which the nitroxide is reduced by vitamin C. Such a spatial proximity to the probe of remote residues in the protein sequence suggests an overall folding of tau protein in its native monomeric form such as the paperclip-like conformation. **(B)** FRET measurement of distances requires both the mutagenesis of a hydrophobic amino acid into tryptophan as FRET donor probe (red spot) and coupling of IAEDANS as FRET acceptor probe (blue spot) to either a native (C322) or engineered cysteine (T17C) of tau protein. Different combinations of positions of FRET donor and acceptor probes allowed to define the paperclip conformation of native monomeric tau where both N- and C-termini are in close proximity of the central MTBR functional domain (Jeganathan et al., 2006; Jeganathan et al., 2012).

The measurement of distance between FRET pairs in tau mutants carrying a tryptophan and a AEDANS-labeled cysteine, both residues being introduced by site-directed mutagenesis highlighted a paperclip-like conformation in native, soluble tau protein where both N- and C-termini fold over the MTBR (Figure 9B). Conformational changes of the paperclip preferential dynamic fold was observed upon phosphorylation and denaturation (Jeganathan et al., 2006; Jeganathan et al., 2012). The global fold was further confirmed by paramagnetic relaxation enhancement (PRE) NMR signals (see here below). The synthesis and FRET analysis of a site-specific, dual-labeled Htt exon1 indicated a progressive compaction of the protein upon increasing polyQ length in contrast to the pathological threshold length associated to HD (Warner et al., 2017).

Different conformational ensembles of α-synuclein were investigated inside cells using FRET pairs of fluorophores. FRET measurements allow to distinguish the unstructured α-synuclein and the α-helical conformation when α-synuclein is bound to the membrane, as both conformations are characterized by differences in the distance between both labels, the distance between the labeled amino-acid position being larger in the unstructured form (Fakhree et al., 2018). Introduction of labels can be coupled to introduction of modifications, using combination of the strategies here above briefly described using strategies addressed in paragraph 2. The semisynthesis of pY39 α-synuclein allowed to include a pair of FRET fluorescent labels using 1) coupling to cysteine and 2) UAA (propargyl-tyrosine, PpY) incorporation by amber codon suppression followed by click chemistry to reveal that phosphorylation of Y39 primarily acts on aggregation with only small interference with monomer conformation (Pan et al., 2020).

Processing of APP to generate the Aβ peptide was elegantly investigated in cells using a dual labeling scheme (van Husen et al., 2019). The APP C-terminal tail was labeled using a SNAP-tag while an UAA (trans-cyclooctene lysine) was introduced at a specific location in APP, corresponding to the Aβ peptide, by stop codon suppression. The side-chain of this UAA was next modified to attach a fluorescent dye (6-methyl-tetrazine-BODIPY-FL) by click chemistry reaction, while the unnatural residue was functionalized using TMR-Star. This double-labeling scheme allows to follow APP processing into a C-terminal fragment and Aβ peptide, and imaging the trafficking of the APP cleavage products in live cells.

Some *in vivo* experiments take advantage of the specific pH that characterizes some subcellular microenvironments to track the processing of amyloid proteins once they enter the cells. For example, CPX azide derivative was attached on α-synuclein using the UAA PpY functionalized by an azide-alkyne cycloaddition in a click chemistry approach. The labeled α-synuclein incorporated in fibrils was then tracked over time after it had entered neurons. Aggregates penetrating endosomes were highlighted by the green to red emission shift of CPX in that specific compartment (Jun et al., 2019).

Site-directed spin labeling, coupled to nuclear magnetic and/or electron paramagnetic resonance (EPR) measurements, are also of interest to study conformational fluctuations of amyloid proteins. A commonly employed paramagnetic label is the nitroxide moiety, which harbors an unpaired electron. This label is small and expected to cause no conformational perturbation of the functionalized protein. The paramagnetic effect of the electron is indirectly detected by NMR upon perturbation of the recorded signals due to enhanced transverse relaxation rate induced by proximity to the label (Clore et al., 2007). This effect has a r^−6^ dependence on the electron-proton distance (r) and thus allows the detection of long-range interactions in proteins (Figure 9A). To determine an ensemble of conformations consistent with PRE measurements, NMR signal intensity ratio (with/without nitroxide effect) can be converted into distance restraints. Direct measurements by EPR spectroscopy give information on the dynamics of the probe environment and is a convenient way to track oligomer formation (Zurlo et al., 2019). Double nitroxide labeling have interesting applications in both cases. Pulsed double electron–electron resonance (DEER) EPR on frozen samples can be used to derive a distribution of distances between the paramagnetic probes. For NMR applications, paramagnetic relaxation interference (PRI) in the presence of two nitroxide probes has been proposed to detect concerted conformational fluctuation in disordered proteins (Kurzbach et al., 2016). PRI being sensitive to correlated motions in the protein can help to detect a sparsely populated state in the ensemble, which nevertheless can be significant on the aggregation pathway. The Overhauser dynamic nuclear polarization (DNP) made use of site-specific nitroxide spin label attachment to monitor electron-spin interactions with water molecules and detect localized perturbations of hydration during tau protein aggregation related to the magnetic dipolar nature of interactions between the nitroxide unpaired electron and water protons that are mostly localized within 5Å. This allows discriminating hydration changes upon the formation of organized protein-protein interfaces associated to fibrils from non-specific protein-protein interactions (Pavlova et al., 2009).

Kinetics of α-synuclein aggregation was followed by continuous wave (CW) EPR using nitroxide spin-labeling to monitor oligomer formation/disappearance and how the aggregation pathway develops. The CW-EPR method uses the rotational diffusion time of the spin-labeled protein, visualized as EPR lineshapes, to cover the nanosecond to second time scales. The label dynamics is influenced by the local structure and macromolecular interactions at proximity of the probe. The MTSL label ((1-oxyl-2,2,5,5-tetramethylpyrroline-3-methyl)-methanethiosulfonate) was attached on α-synuclein C56, introduced by site-specific mutagenesis. The monomer, oligomer and fibril concentrations were evaluated at different time points during kinetics experiment, based on mobility differences of the species (Zurlo et al., 2019). Pulsed EPR measurements of various amyloid fibrils were also performed to gain structural information (Bedrood et al., 2012; Siddiqua et al., 2012; Pornsuwan et al., 2013).

Interestingly, EPR is also amenable to *in vivo* experiments (Cattani et al., 2017). Intracellular CW-EPR combined to a systematic spin-labeling scan of micro-injected recombinant α-synuclein shows that the majority of α-synuclein remains in monomeric, intrinsically disordered state in the *Xenopus laevis* oocyte cells, even in the case of disease variants A30P or A53T that are correlated with PD familial forms. These latter variants show an increased rate of oligomerization and decreased membrane binding (Flagmeier et al., 2016). The spin label for this *in-cell* study is 3-maleimido-PROXYL, which is more stable in the reducing environment of the cell than MTSL.

PRE of NMR signals were used to obtain structural information on α-synuclein. Three MTSL nitroxide labels were attached to single cysteine mutants. Measurements showed that α-synuclein adopts in its native state an ensemble of conformations that is stabilized by long-range interactions. These interactions are characterized by contacts between the central and C-terminal domains that shield the aggregation-prone non-amyloid-β component (NAC) (Bertoncini et al., 2005a; Dedmon et al., 2005). PRE of NMR signals additionally show that the familial PD mutations, A30P and A53T, perturb the ensemble of α-synuclein conformations pointing toward a reduced shielding of the hydrophobic NAC region in the A30P and A53T mutants of α-synuclein (Bertoncini et al., 2005b).

PRE of NMR signals were also used to build tau conformational ensemble. The paramagnetic probe MTSL was attached on the native cysteine residues, or on five single-cysteine mutants located in the various tau domains (Figure 9A). The intricate network of transient long-range interactions confirms the “hairpin model” of tau dynamic conformation. Interactions of the spin-label with distinct area of the tau sequence indeed show that the C-terminal domain transiently interacts with both the PRD and the N-terminus. The conformational dynamics of tau wild-type or harboring the P301L familial FTDP mutation were additionally compared using a combination of PRE and PRI experiments (Kawasaki and Tate, 2020). PRI data demonstrate alteration of the short- and long-range correlated motions in P301L tau mutant, promoting transient exposure of the PHF6 motif that plays a role as nucleus of tau aggregation (Kawasaki and Tate, 2020).

#### 4.1.4 Positron Emission Tomography Imaging Tracers

Brain imaging is a major area for which chemical biology has made a crucial contribution to support research on disease mechanisms in central nervous system proteinopathies and to deliver promising compounds for clinical use by providing tracers for *in vivo* imaging using positron emission tomography (PET) (Ni and Nitsch, 2022). This topic can be here only briefly covered given its scope. The challenge in this field is to provide specific and sensitive tracers reaching the brain, based on ^18^F, ^11^C or ^3^H radio-compounds. Ideally, the tracers should allow early and differential detection of diagnostic biomarkers before the appearance of clinical symptoms to facilitate access to treatment ahead of the irreversible damages due to neuronal and synaptic connectivity loss.

**FIGURE 10:**
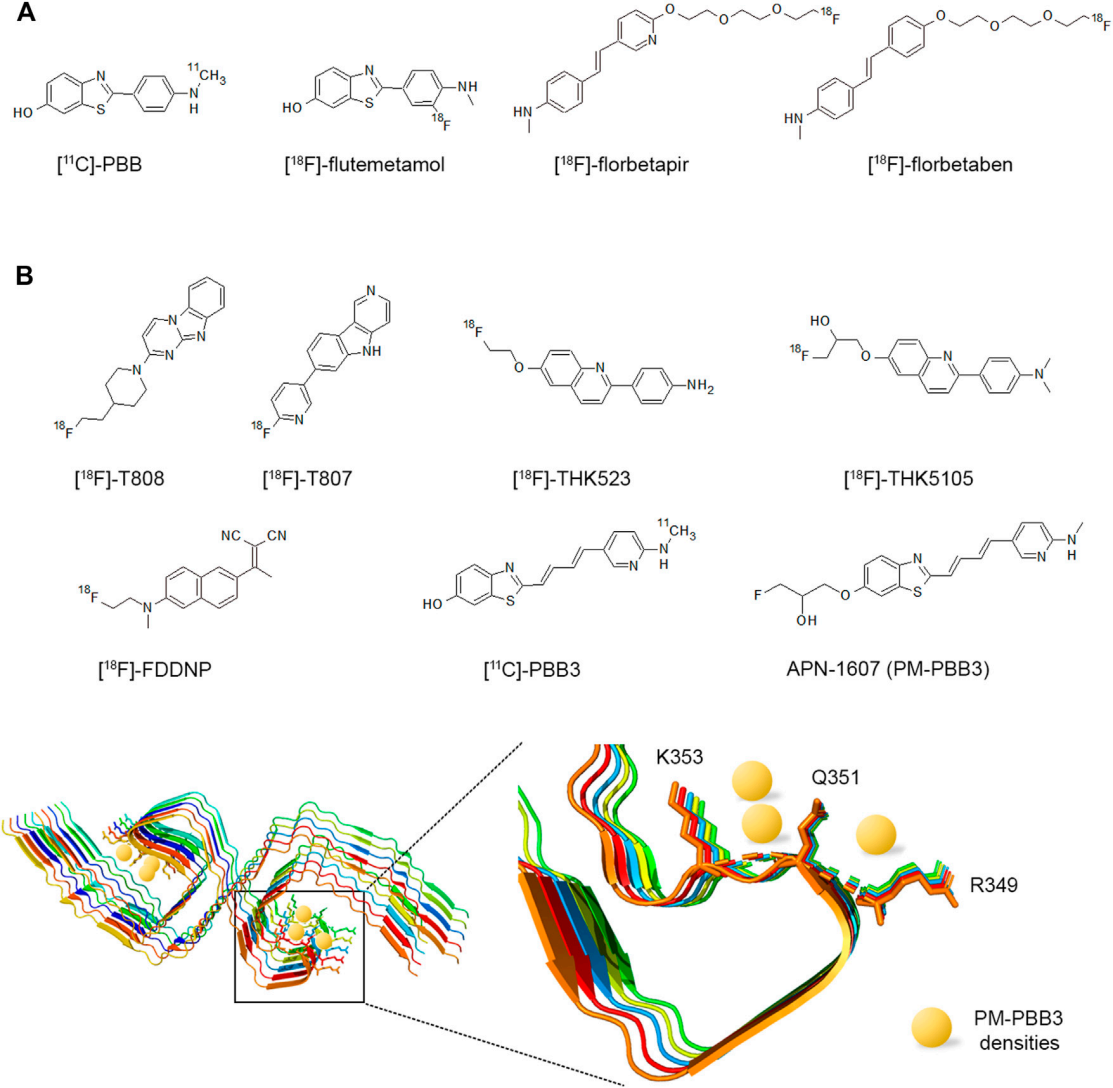
Structures of PET radiotracers for amyloid imaging of Aβ deposits **(A)** or tau inclusions **(B)**. The cryo-EM structure of the PM-PBB3 (APN-1607) compound bound to PHF-tau amyloid structure shows selected interactions in the C-shaped cavities with R349, Q351 and K353 amino acids of the tau MTBR R4 repeats (PDB ID: 7NRV).

Imaging of AD brain deposits started with the radio-compound [^11^C]Pittsburgh compound B (Klunk et al., 2004) that derives from ThT to detect β-amyloid deposits by PET imaging (Figure 8B). Amyloid PET is based on β-sheet structure detection and compounds are mainly benzothiazole and benzoxazole derivatives. Among these tracers, three have already been approved for clinical use: florbetapir (Clark, 2011), flutemetamol (Curtis et al., 2015) and florbetaben (Figure 10A) (Sabri et al., 2015). Six binding sites on Aβ fibrils are proposed for the tracers, based on molecular modeling studies. Binding sites can be divided between core sites (buried in the fold with low solvent accessibility), and surface binding sites, the latter providing less specificity (Murugan et al., 2016). These *in silico* studies suggest that amyloid tracers of different structures could detect different Aβ fibrils.

Tau-specific ligands have also been developed for clinical use, and enable *in vivo* PET imaging of tau deposition (Leuzy et al., 2019; Saint-Aubert et al., 2017). Monitoring tau deposits is of interest for diagnosis as cortical retention of tau tracers better correlates with cognitive decline than Aβ deposits (Pontecorvo et al., 2017). These small molecules are however specific for the β-sheet structure adopted in tau fibers, and not to tau protein *per se*. This structure is found in other proteinopathies that might co-exist, which can complicate the image interpretation. Tau tracers should not only be able to cross the blood brain barrier, but additionally penetrate the intracellular compartment where tau fibrils also reside. Tau tracers belong to three major different chemical families, namely, pyridinyl-butadienyl-benzothiazole derivative (^11^C-PBB3) derived from the same family as Pittsburgh compound B, benzimidazole-pyrimidine derivatives (^18^F-T807/AV 1451, and ^18^F-T808) as well as different arylquinoline derivatives (^18^F-THK5105, ^18^F-THK523, ^18^F-THK5117, and ^18^F-THK5351) (Figure 10B). These tracers bind to tau with affinities in the nanomolar-picomolar range. These first-generation tracers are however reported to bind to a number of off-targets such as monoamine oxidase-B in the basal ganglia (Lemoine et al., 2017). It leads to optimization of the tracer binding properties and delivered a second-generation of tracers with better specificity and a broader dynamic range. Based on *in vitro* binding assays, three different high affinity binding sites have been proposed in tau fibrils (Figure 10B). Computer-assisted docking of the tracers based on the cryo-EM structures of tau fibrils predict four different binding sites for the various tracers (Murugan et al., 2018; Kuang et al., 2020; Murugan et al., 2021).

The tau tracer AV-1451 (Johnson et al., 2016), also known as flortaucipir, specifically binds to tau fibril from AD and non-AD tauopathies (Lowe et al., 2016). The clinical validity of flortaucipir has been demonstrated as diagnostic biomarker and it is approved for imaging tauopathy in AD patients. A study based on PET imaging with [^18^F]AV-1451 confirms that tau pathology can propagate though a neuron-to-neuron transfer (Cope et al., 2018). The different types of tau deposits in the different diseases, highlighted at the atomic level by the cryo-EM structures (Lippens and Gigant, 2019), made it quite challenging to develop tau PET tracers. Given the heterogeneity in tau strains, tracers could be developed that recognize a sub-set of such strains. This strain heterogeneity, not only between pathologies but also between individual patients, is highly relevant because it is proposed to be linked to clinical heterogeneity in patients with typical AD (Dujardin et al., 2020). These *in vivo* diagnostics tool compounds will have huge impact on future assembly of better characterized cohorts and provide an adequate monitoring in clinical assays of the treatment effects.

### 4.2 Chemical Methods for Deciphering the Amyloid Aggregation Pathway

#### 4.2.1 Keeping Amyloids Under Control With Chirality and Molecular Switches

**FIGURE 11:**
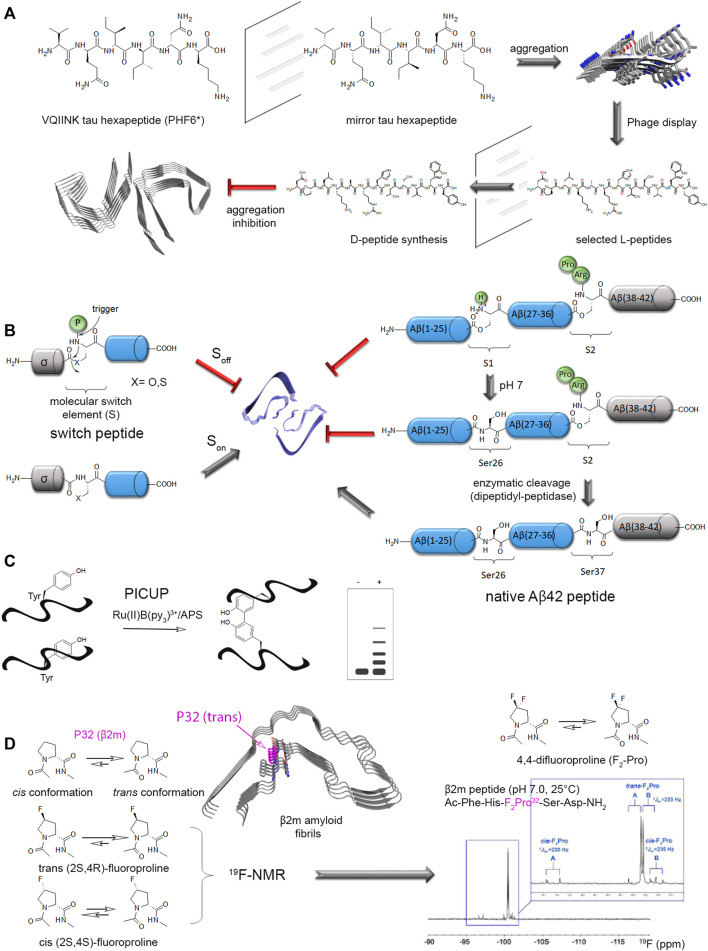
Chemical biology tools for controlling and deciphering the amyloid aggregation pathways. **(A)** Fibrils made of the mirror PHF6* hexapeptide of tau consisting of D-enantiomeric amino acids was used in mirror image phage display to screen L-peptides that bind to mirror PHF6* fibrils. The mirror peptides of the selected L-peptides were synthesized and evaluated as inhibitors of full-length tau aggregation exhibiting protease stability and reduced immunogenicity (Dammers et al., 2016; Malhis et al., 2021). **(B)** The strategy of “click” peptides or “switch” peptides is used to control protein aggregation and decipher the molecular elements responsible of amyloid aggregation (left panel). A molecular switch element (S) is under control of a protecting group “P” (green dots) that prevent aggregation (S_off_) and is cleaved by an appropriate trigger factor (pH, enzyme, reducing agents, light, …). Upon deprotection, the spontaneous O→N or S→N acyl shift within the molecular switch element restores the native peptide bond between both fragments at its N- and C-terminal sides. As a result, the structural induction unit σ (grey fragment) is linked through a native peptide bond to the remaining part of the protein (blue fragment). If the deprotection and subsequent acyl shift trigger the amyloid aggregation of the protein of interest (S_on_), σ is identified as an aggregation hot spot as exemplified by the C-terminal region of Aβ42 peptide (right panel). In this case, a first molecular switch element (S1) at S26 under control of pH is removed without triggering any fibril formation. The enzymatic cleavage of the second switch element (S2) by the dipeptidyl-peptidase at S37 enables the fibrillization of native Aβ42 peptide supporting a role of the C-terminus in conformational changes and aggregation. **(C)** The photo-induced cross-linking of unmodified protein (PICUP) strategy is used to generate protein oligomers by light irradiation of a photocatalyst, the tris(bipyridyl)ruthenium(II), in presence of ammonium persulfate. Metastable oligomers that form transiently along the amyloid aggregation pathway, as shown by polyacrylamide gel electrophoresis under denaturing conditions, can thus be captured for further structural and functional investigations. **(D)** The use of fluoroproline derivatives, 4-cis-fluoro, 4-trans-fluoro or 4,4-difluoro-proline, in controlling amyloid protein conformational changes and aggregation rely on the alteration of the cis/trans conformational exchange rate and equilibrium of the peptidyl-prolyl bond with the 4-cis-fluoroproline the most favorable to the cis amide bond conformation. Furthermore, fluoroproline can be involved as probes in ^19^F-NMR studies of conformational exchange in peptides and proteins as shown for a model peptide of β2m, Ac-FH(F_2_-P^32^)SD-NH_2_. The ^19^F-NMR spectrum of the β2m peptide containing a 4,4-difluoroproline was reproduced from reference (Torbeev and Hilvert, 2013). The corresponding sequence is shown as sticks in the structure of β2m amyloid fibrils (PDB ID: 6GK3) with P32 (magenta) in trans conformation.

Manipulating amino acid chirality into amyloid sequences has been implemented through SPPS to reveal amyloidogenic pathways and critical hot spots in amyloid assembly (Foley and Raskatov, 2021). In a strategy termed “chiral editing”, d-amino acid (D-AA) enantiomers that are site-specifically introduced either at selected positions of interest or randomly by D-AA scanning proved to be useful tools to highlight mechanistic details of Aβ aggregation and toxicity, as well as key residues in this process. Mirror peptides of Aβ42 and Aβ40 that incorporate D-AA along the entire sequence were shown to accelerate Aβ fibrillization into nontoxic fibrils in a racemic mixture with L-Aβ peptide (Dutta et al., 2017). The chiral inactivation of Aβ generates distinct fibrillar structures likely through the formation of rippled instead of pleated cross-β sheets (Dutta et al., 2019a). D-Aβ42 oligomers were also shown to have a reduced, if any, cytotoxicity and cellular internalization (Dutta et al., 2019b). Stereoselective interactions with chiral components of the phospholipid membrane could be responsible for this differential effect. Overall, the advantage of D-AA and mirror peptides is that chiral mutations do not change the physicochemical properties of the peptides (side chain chemical groups, size, polarity, charge…). It was shown that D-peptides targeting the mirror VQIINK tau hexapeptide (PHF6*) fibrils inhibit full-length tau aggregation while exhibiting protease stability and reduced immunogenicity (Figure 11A) (Dammers et al., 2016; Malhis et al., 2021). Based on these properties, D-peptides may be useful as therapeutic and diagnostic agents in amyloid-related diseases (Abdulbagi et al., 2021).

It is well documented that some amyloid-forming sequences spontaneously undergo into conformational changes and aggregate during SPPS, purification and other processing steps. Keeping amyloids under control with molecular switches is an efficient strategy to decipher their mechanism of aggregation and toxicity by controlling the onset of aggregation at early stages of initiation/nucleation, and identifying aggregation hot spots in amyloid sequences (Butterfield et al., 2012). A molecular switch is defined as a reversible modification at a selected location within the peptide sequence that tunes on and off the amyloid aggregation properties. O→N (or S→N) acyl shift of isopeptide bonds in so-called “click peptides” or “switch peptides” are usual molecular switches that are under control of a protecting group (Figure 11B). The native peptide bond is restored by O→N or S→N intramolecular rearrangement once the protection is released. The introduction of O-acyl or S-acyl isopeptide bonds efficiently disrupt misfolding and oligomerization of amyloid-forming sequences. In some instances, the release of an inhibitory element elicits amyloid assembly. A panel of triggering factors (pH change, enzymatic reactions, reducing agents, photoreactions) can be used for this purpose allowing a rapid switch from the inert into aggregation-prone sequence as conveniently illustrated for small, synthetic Aβ, IAPP or PrP peptides (Taniguchi et al., 2009; Taniguchi et al., 2006; Bosques and Imperiali, 2003). This provided information into the fine molecular mechanisms of Aβ42 (dis)aggregation by decoupling aggregation-prone sequence from a nucleating or structural element to prevent aggregation or, reversely, by favoring disassembly of amyloid fibrils into a soluble conformation (Figure 11B) (Dos Santos et al., 2005; Mimna et al., 2007). These methods also found applications in the screening of anti-aggregation compounds (Sohma et al., 2011).

### 4.2.2 Stabilizing Oligomers by Photo-Induced Cross-Linking

Often poorly defined at the molecular level, oligomers are of variable size ranging from dimers to a few hundred monomers. Their molecular and structural characterization suffers from their unstable nature and heterogeneity, and must overcome methodological hurdles. Cross-linking has been used to stabilize Aβ dimers in several experimental setup (Bitan and Teplow, 2004) although covalently cross-linked Aβ dimers isolated from AD brains were found to be neurotoxic species of biological relevance (Vázquez de la Torre et al., 2018; Brinkmalm et al., 2019). Cross-linking applied to tau protein has shown conformational differences between inert and seed-competent monomer highlighting inaccessibility vs. exposure, respectively, of the hydrophobic, amyloid-prone PHF6 and PHF6* sequences (Mirbaha et al., 2018). The photo-induced cross-linking of unmodified proteins (PICUP) method by light irradiation of a tris(bipyridyl)ruthenium(II) photocatalyst was amenable to the study of amyloid proteins to capture intermediate, metastable oligomers for further purification, characterization and investigation of their seeding activity in inducing fibril formation (Figure 11C) (Bitan et al., 2001). The site-specific incorporation of photo-induced cross-linking probes such as trifluoromethyldiazirine derivative of phenylalanine into the sequence of Aβ peptide fragment stabilized “on-pathway” oligomeric species providing structural details about the very first pathological species involved in fibrillization onset (Smith et al., 2008).

### 4.2.3 Fluorine Labeling and ^19^F NMR

^19^F NMR offers an interesting opportunity to study fibril formation, as it provides residue-level quantitative information about structure and mechanism. Because the ^19^F nucleus is highly sensitive to its chemical environment, it displays a very wide range of chemical shifts and chemical shift perturbations even for very subtle structural changes. Given also the complete absence of ^19^F in biomolecules, no background signals are present, meaning conformational changes can be detected using simple 1D spectra. A wide repertoire of fluorine labelled amino acids exists, and can be incorporated in recombinant proteins or via chemical peptide synthesis (Salwiczek et al., 2012; Gimenez et al., 2021). ^19^F NMR spectroscopy is able to detect signals from soluble proteins or small soluble aggregates, conversely to ThT that detects fibrils. Fluorotyrosine-labelled α-synuclein was used to monitor aggregation kinetics by acquisition of one-dimensional spectra (Li et al., 2009). These kinetics data were compared with a fluorescence-monitored time course, in the presence of ThT, in the same conditions. The time course of the ^19^F NMR data was similar to the ThT-monitored aggregation kinetics. ^19^F NMR allows the additional quantification of the fraction of monomers or small oligomers remaining in solution at all time-points during the kinetics. Tracking amyloid formation of IAPP using this technique shows that IAPP fibrillizes without formation of nonfibrillar intermediates, in contrast to the well-studied Aβ and α-synuclein proteins (Suzuki et al., 2012). Interestingly, aggregation can be monitored in this manner without addition of an external reporter, such as ThT. This is of importance when studying inhibitor compounds of the aggregation process. It was indeed shown that ThT strongly competes with a polyphenolic compound for binding sites on IAPP fibers. To investigate the conformational polymorphism of the prion oligomers, a 3-fluoro-phenylalanine reporter was introduced in the prion fragment PrP(90–231). ^19^F-NMR spectroscopy was used to quantify the populations of oligomeric species on the path from the monomeric soluble PrP state to fiber formation. In addition, thermodynamic and kinetic parameters of the interconversion of the oligomeric species were extracted from the ^19^F NMR data obtained by temperature or pressure scanning (Larda et al., 2013). ^19^F NMR real-time measurements was also used to investigate the formation of small oligomers during the aggregation of Aβ1−40. Five distinct oligomers (estimated of 30–100 kDa) with unique spectral signatures, which allow to monitor each individually, were detected at distinct time points in the time-course of fibril formation (Suzuki et al., 2013). Their build-up and decay were evaluated in real-time during aggregation and transient species could be identified. Fluorine can also be used to subtly alter the conformational preference of individual amino acids, allowing their role in the aggregation process to be interrogated. A well-known example is fluorinated prolines, which, besides allowing ^19^F NMR investigation (Sinnaeve et al., 2021), also modulates the proline *cis*/*trans* ratio and the interconversion rate (Figure 11D) (Verhoork et al., 2018). The human protein β2-microglobulin (β2m) aggregates as amyloid fibers in patients undergoing long-term hemodialysis. Isomerization of P32 from its native *cis* to a nonnative *trans* conformation was shown to cause β2m misfolding and aggregation using replacement of this crucial residue with 4-fluoroprolines by total chemical synthesis (99 residues) (Figure 11D). The β2m monomer was stabilized by incorporation of (2S,4S)-fluoroproline (or *cis*-fluoroproline), which favors the native *cis* amide bond while the monomer was destabilized by the (2S,4R)-fluoroproline (or *trans*-fluoroproline), which disfavors *cis* conformation (Figure 11D). 4,4-difluoroproline, which enhances the isomerization rate without modifying the *cis*/*trans* equilibrium relative to regular proline, increases the oligomerization rate. Altogether, these data demonstrate the major effect of P32 conformation on β2m aggregation (Torbeev and Hilvert, 2013). To study the role in the aggregation process of a kink within the R3 repeat of the tau protein, at residue S316, a peptide was used containing a S316P mutation (Jiji et al., 2016). In the peptide wherein (2S,4S)-fluoroproline at position 316 was introduced, the *cis* conformation is favored compared to regular prolines and the modified peptide aggregates twice as fast. In the peptide containing the stereoisomeric (2S,4R)-fluoroproline at position 316, the *trans* conformation is preferred and the modified peptide aggregates at a slower rate. The results clearly showed that favoring the cis conformer of L315-P316 peptide bond promotes aggregation of the R3-S316P peptide. The authors of this study propose that a *trans*-to-*cis* conformation shift in the peptide bond preceding P316 occurs during peptide aggregation, with the potential type I β-turn encompassing P316 being replaced by a type VI β-turn favored by the *cis* bound in the R3-S316P fibrils.

Taken together, these various examples illustrate that the introduction of fluorine can be very useful in deciphering key aspects of the aggregation process by directly reporting events at a residue scale. This allows tracking of conformational changes occurring on the path to fibril formation and documenting the conformational heterogeneity due to the formation of oligomeric species using ^19^F NMR, but equally can be used to elucidate the role of individual residue conformations during aggregation.

### 4.3 Nanobody-Directed Detection and Modulation of Amyloid Proteins

Single domain antibody fragments, scFv (single chain variable fragments), and VHHs (heavy chain variable domain or single-domain antibodies or nanobodies) are interesting tools to probe protein aggregation (De Genst et al., 2012; Pain et al., 2015). These single domain antibodies have many interesting properties such as their low molecular weight and their ease of production, which makes them perfect candidate for protein engineering (Holliger and Hudson, 2005; Deffar et al., 2009; Desmyter et al., 2015; Könning et al., 2017).

*In vitro*-based ligand selection, without the use of animal immunization, made it possible to identify antibody fragments with a conformational selectivity. Antibody fragments can thus be selected to target each species formed on the way of fibril formation, from the soluble monomer to the fibril structure. Their ease of expression and small size in particular allow them to be used efficiently in combination with NMR spectroscopy and X-ray crystallography. Single domain antibodies have been reported to specifically detect soluble oligomers of various amyloidogenic proteins (Emadi et al., 2009; Zhang et al., 2011; Butler et al., 2016). They can thus be used to address the biophysical properties/cytotoxicity relationship of the oligomers, which is a challenging task given their transient and heterogenous nature (Bitencourt et al., 2020). Antibody fragments are also helpful to decipher the underlying microscopic stages of the aggregation mechanism (De Genst et al., 2012; Pain et al., 2015). NUsc1, for an example of scFv, recognizes a unique conformational epitope displayed on oligomers of Aβ and preferentially select for oligomers larger than 50 kDa that are neurotoxic (Sebollela et al., 2017). NUsc1 discriminates oligomers over monomers or fibrils. Interestingly, rational design was used to obtain conformation-specific single domain antibodies able to detect Aβ oligomers (Aprile et al., 2020; Limbocker et al., 2020). These antibody fragments were designed to bind different epitopes covering the entire sequence of the target protein. This procedure enables the determination through *in vitro* assays of the regions exposed in the oligomers but not in the fibrillar deposits. VHHs that recognize α-synuclein fibers at different maturation stages have also been characterized and used to gain insight in fibril structures (Guilliams et al., 2013), thus helping in deciphering the fibril formation mechanisms. Even antibody fragments that bind the monomeric soluble forms of amyloidogenic proteins can be used to provide information on the aggregation process. For example, scFv antibody D10, which binds α-synuclein monomers in the C-terminal part, was instrumental in showing that this region of α-synuclein interferes in the aggregation process (Zhou et al., 2004). The nanobodies NbSyn2 and NbSyn87 have similarly been used to identify the role of different C-terminal regions of α-synuclein in aggregation (De Genst et al., 2010; El-Turk et al., 2016). Finally, a VHH targeting α-synuclein showed protection against α-synuclein toxicity in a cellular model. This study also showed that a proteasome targeting PEST motif enhanced this protective effect (Butler et al., 2016). The use of VHH in tauopathies is also considered: a VHH directed against the PHF6 motif of tau, a nucleation core, proved to be efficient against tau fibril formation in *in vitro* and cellular models, and showed efficacy in preventing tau seeding in a mouse model (Dupré et al., 2019; Danis et al., 2022).

**FIGURE 12:**
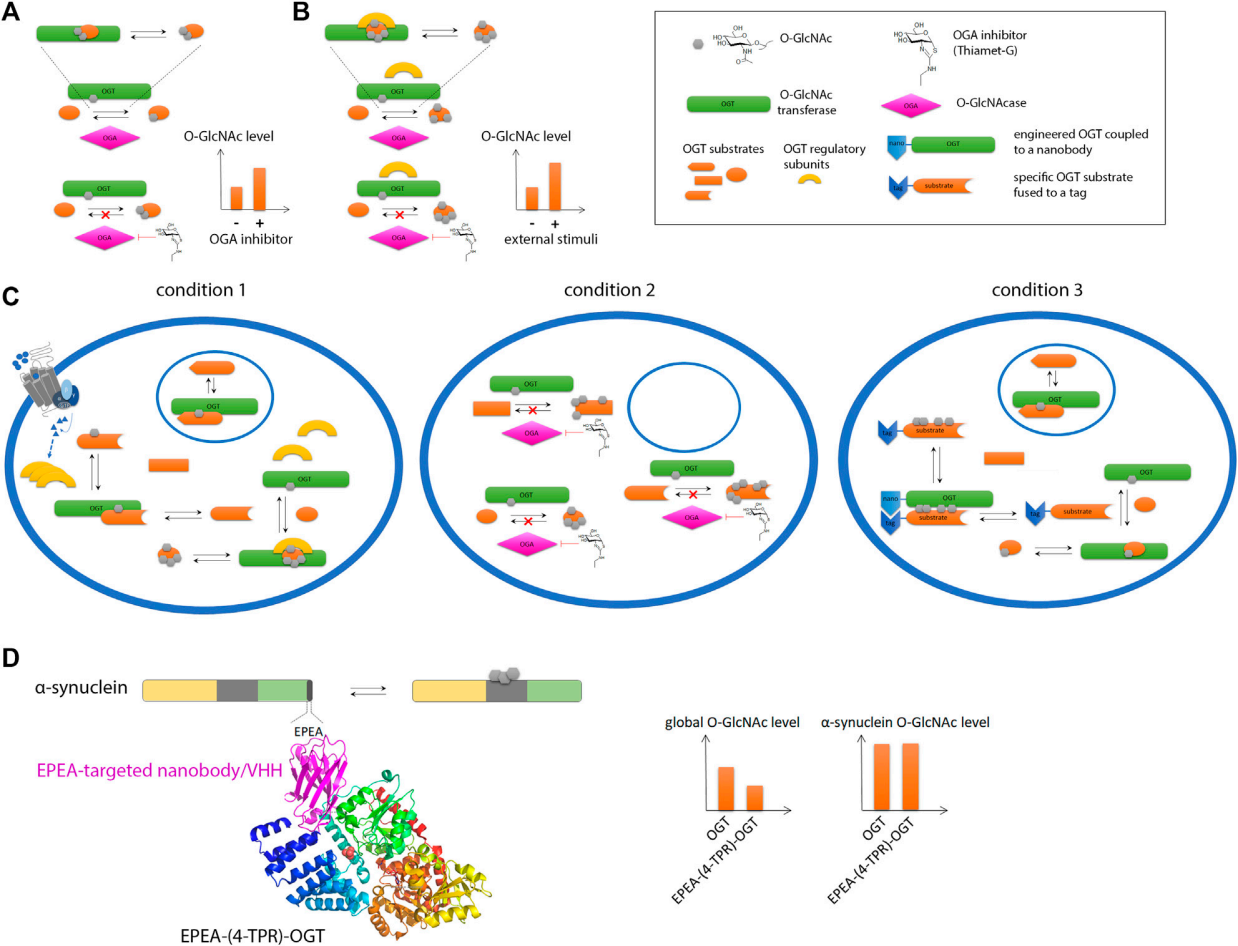
Modulation of O-GlcNAc modification of proteins by proximity-induced protein glycosylation using nanobody-OGT protein engineering. **(A)** Schematic representation of the O-GlcNAc dynamics regulated by single OGT and OGA enzymes, and modulation of OGA activity by an OGA inhibitor such as Thiamet G contributing to overall increased O-GlcNAc levels. **(B)** As OGT is the unique O-GlcNAc transferase in human, it has been suggested that it may act with regulatory subunits that address OGT to specific substrates depending on external stimuli and increase protein-specific O-GlcNAc levels. Treatment with Thiamet-G by increasing overall O-GlcNAc levels may contribute to increase protein-specific O-GlcNAc level. **(C)** Modulation of protein O-GlcNAc levels in cell or animal models. Condition 1, external stimuli and signal transduction lead to the expression of protein-specific regulatory subunits of OGT that increase its activity on selected proteins; condition 2, overall increase of protein O-GlcNAc levels upon treatment with Thiamet-G; condition 3, selected increase of O-GlcNAcylation of a target substrate by a nanobody fused to OGT targeting a specific protein tag. **(D)** This latter method (C, condition 3) was further extended to O-GlcNAcylation of endogenous α-synuclein using a nanobody-OGT targeting α-synuclein EPEA C-terminal sequence (Ramirez et al., 2020). EPEA-(4-TPR)-OGT refers to a truncated form of OGT containing 4 tetratricopeptide repeats fused to a nanobody targeting the EPEA sequence of α-synuclein. The TPR truncation of OGT limits the overall increase of O-GlcNAc levels while OGT coupling to a nanobody targeting the EPEA sequence increases the selectivity for α-synuclein. This approach leads to a selective elevation of α-synuclein O-GlcNAc levels.

Besides nanobodies directly targeting the amyloid-forming regions in proteins, other routes involved in the regulation of amyloidogenic properties such as functional modulation by PTMs may be addressed with nanobodies (El Turk et al., 2018). As a PTM involved in regulation of protein functions, the *O*-GlcNAc modification started to be scrutinized in the processes of amyloid formation (Ryan et al., 2019). To achieve this goal, global alteration of *O*-GlcNAc levels can be fulfilled with chemical inhibitors (Yuzwa et al., 2008; Yuzwa et al., 2012; Yuzwa et al., 2014b; Gloster et al., 2011; Dorfmueller et al., 2006; Macauley et al., 2005; Martin et al., 2018; Haltiwanger et al., 1998; Macauley and Vocadlo, 2010) (Figures 12A–C) or through manipulating gene expression of enzymes involved in the hexosamine biosynthesis pathway (HBP) (Wells et al., 2003) or directly the *O*-GlcNAc cycling enzymes, OGT and OGA (Akan et al., 2018; Wang et al., 2012; Shafi et al., 2000; O'Donnell et al., 2004; Olivier-Van Stichelen et al., 2017). Alternatively, approaches limiting an overall elevation of *O*-GlcNAc levels in cells are of great interest (Gorelik et al., 2019; Ramirez et al., 2020). Along these lines, nanobodies fused to OGT were engineered as proximity-directing agents in an innovative strategy for selectively increasing *O*-GlcNAc levels on a target protein in cells aiming at understanding the role of this key PTM (Figure 12C) (Ramirez et al., 2020). Truncation of the tetratricopeptide repeat (TPR) domain of OGT which is implicated in protein-protein interactions for substrate recognition and binding further achieved increased nanobody-directed selectivity for the target protein (Ramirez et al., 2020). First attempts were made with nanobodies targeting protein tags excluding the modification of endogenous proteins but providing increased levels of *O*-GlcNAcylation with similar O-GlcNAc profiles as OGT (Figure 12C). Moreover, this approach represents a versatile and economical way of increasing *O*-GlcNAc levels of a specific protein by preventing from screening nanobody libraries against the protein of interest. The use of a α-synuclein-targeted nanobody-OGT further proved its efficiency by selectively modifying endogenous α-synuclein in a cell model (Figure 12D). This approach could be further extended to *in vitro* systems to improve the efficiency of O-GlcNAc installation. A similar strategy for targeted protein deglycosylation in cells was described using nanobody-fused split OGA (Ge et al., 2021). As OGT TPR truncation reduces the overall elevation of protein O-GlcNAcylation, splitting of OGA minimize the overall effect of OGA overexpression on the O-GlcNAcome. Both nanobody-fused OGT and OGA are informative, complementary approaches to decipher O-GlcNAc-mediated functional regulation *in vivo* and in cells. Other strategies of proximity-induced O-GlcNAc modulation were evolved albeit not applied to amyloid proteins by now. They are based on designing recombined RNA aptamers that bind to both OGT and the protein of interest thereby inducing proximity for targeting OGT to specific proteins in cells (Zhu and Hart, 2020).

An extension of the antibody fragment properties in tracking amyloid formation could be *in vivo* diagnosis to track disease progression. VHHs are well considered in imaging the brain on multiple targets (Li et al., 2016), and coupling with the possibility to engineer them to cross the blood brain barrier could lead to a whole new series of molecule in diagnosis of brain pathologies. VHHs could well be the new trend in brain diagnostic tools, as many studies have shown their potency, as in amyloid plaques imaging (Li et al., 2016; Vandesquille et al., 2017; Pothin et al., 2020).

These different examples give a short glimpse at the power of antibody fragments targeting various forms of amyloid proteins. They can be useful research tools, easy to manipulate and engineer, to help in deciphering complex mechanisms down to a molecular basis. Coupled to the progresses in rational design, antibody fragments show additional promises for diagnosis and therapy (Gerdes et al., 2020; Messer and Butler, 2020) and could address the bottle-neck of crossing the blood brain barrier to reach amyloid deposits in the central nervous system.

## 5 Conclusion

The recent resolution of amyloid fibril structures from individual brains at near-atomic resolution by cryo-EM has brought into focus the complexity of amyloid folds and polymorphism associated to neurodegenerative diseases while leaving open many questions. The regulation of amyloid assembly and selection of polymorph by PTMs of amyloid proteins and their interacting cofactors still remains to be deciphered as they are only poorly described in the cryo-EM structures due to heterogeneity. Chemical biology is helping to delineate the intricate processes of amyloid fibril formation and propagation, and amyloid protein toxicity. The tool kit from chemical biology and protein engineering offers handling of site-specific, posttranslationally modified amyloid proteins by semisynthesis, genetic code expansion, posttranslational chemical mutagenesis, or combination thereof. It also provides specific tools for the detection and characterization of amyloid PTM codes including enrichment strategies. In conjunction with cryo-EM, it should afford new insights into the role of PTMs in the mechanism of amyloid aggregation and how they shape the amyloid fold. In this regard, this rapidly expanding field also provides new probes to control and unravel the mechanisms of amyloid formation and associated conformational changes as probed by spectroscopic and optical methods, and for the detection of amyloids *in vitro* and *in vivo* as crucial mechanistic and diagnosis tools.
